# A Genome Scale Screen for Mutants with Delayed Exit from Mitosis: Ire1-Independent Induction of Autophagy Integrates ER Homeostasis into Mitotic Lifespan

**DOI:** 10.1371/journal.pgen.1005429

**Published:** 2015-08-06

**Authors:** Ata Ghavidel, Kunal Baxi, Vladimir Ignatchenko, Martin Prusinkiewicz, Terra G. Arnason, Thomas Kislinger, Carlos E. Carvalho, Troy A. A. Harkness

**Affiliations:** 1 Department of Anatomy & Cell Biology, University of Saskatchewan, Saskatoon, Saskatchewan, Canada; 2 Department of Biology, University of Saskatchewan, Saskatoon, Saskatchewan, Canada; 3 Department of Medical Biophysics, University of Toronto, Toronto, Ontario, Canada; 4 Department of Medicine, University of Saskatchewan, Saskatoon, Saskatchewan, Canada; University of California, San Francisco, UNITED STATES

## Abstract

Proliferating eukaryotic cells undergo a finite number of cell divisions before irreversibly exiting mitosis. Yet pathways that normally limit the number of cell divisions remain poorly characterized. Here we describe a screen of a collection of 3762 single gene mutants in the yeast *Saccharomyces cerevisiae*, accounting for 2/3 of annotated yeast ORFs, to search for mutants that undergo an atypically high number of cell divisions. Many of the potential longevity genes map to cellular processes not previously implicated in mitotic senescence, suggesting that regulatory mechanisms governing mitotic exit may be broader than currently anticipated. We focused on an ER-Golgi gene cluster isolated in this screen to determine how these ubiquitous organelles integrate into mitotic longevity. We report that a chronic increase in ER protein load signals an expansion in the assembly of autophagosomes in an Ire1-independent manner, accelerates trafficking of high molecular weight protein aggregates from the cytoplasm to the vacuoles, and leads to a profound enhancement of daughter cell production. We demonstrate that this catabolic network is evolutionarily conserved, as it also extends reproductive lifespan in the nematode *Caenorhabditis elegans*. Our data provide evidence that catabolism of protein aggregates, a natural byproduct of high protein synthesis and turn over in dividing cells, is among the drivers of mitotic longevity in eukaryotes.

## Author Summary

High throughput studies have yielded large collections of genes that together govern *post-mitotic* longevity in eukaryotic cells. However, it is also clear that *mitotic* lifespan is subject to regulation via intricate mechanisms that facilitate exit from mitosis. Elucidating these mechanisms has been the subject of intensive research in part because failure to exit mitosis is associated with cell immortalization, a hallmark of neoplastic growth. Yet, to date mechanisms driving mitotic lifespan remain poorly characterized largely due to the absence of a feasible high throughput screening platform. Here we describe a large-scale screen in yeast *Saccharomyces cerevisiae* for mutants that undergo an atypically high number of cell divisions before exiting mitosis. We report an intricate cross talk between Endoplasmic Reticulum (ER) homeostasis and mitotic longevity. Autophagy, activated in response to ER stress, delays mitotic senescence in part by removing high molecular weight cytoplasmic protein aggregates. This evolutionarily conserved catabolic network similarly extends reproductive lifespan in the nematode *Caenorhabditis elegans*. Our data highlight that, similar to its role in extending post-mitotic lifespan, catabolism of protein aggregates is among the drivers of mitotic longevity in eukaryotes.

## Introduction

Eukaryotic cells undergo a finite number of cell divisions, both *in vitro* and *in vivo*, before irreversibly exiting mitosis [1,2]. Yet single gene mutations can profoundly extend or reduce the number of cell divisions, indicating that mitotic longevity is nevertheless subject to genetic regulation [3–5]. There is now mounting evidence that mitotic senescence is not a default outcome of stochastic cell deterioration, and is instead governed via intricate pathways that in concert limit the number of cell divisions and the onset of complex phenotypes associated with mitotic exit. Elucidating mechanisms that limit the number of cell divisions has been the subject of intensive research in part because failure to exit mitosis is tightly associated with cell immortalization, a hallmark of neoplastic growth [6].

In keeping with the broad conservation of mitotic longevity pathways, the budding yeast *S*. *cerevisiae* has proven remarkably well suited to unravelling molecular mechanisms that govern longevity in eukaryotic cells [3,7]. Large-scale screens of yeast mutants designed to map the underlying longevity networks are reported [4,8]. These screens employed a microdissection assay where daughter cells are successively removed and counted until the mother cells stop dividing. However, this assay is highly laborious and requires several weeks to complete, thus limiting its utility as a high throughput screening method. While a valuable genetic resource in dissecting longevity pathways, many of the emerged mutants currently await validation.

Here we report a high throughput, genome scale screen for isolating mutants with delayed mitotic senescence in yeast. We used the age-dependent loss of transcriptional silencing at the *HML* mating locus [9] to screen a library of 3762 single gene deletions accounting for 2/3 of all yeast annotated ORFs. In parallel to the query library, we similarly screened a control library to search for false positives that display stochastic (not age-dependent) loss of transcriptional silencing. We focused this screen as a positive selection platform for identifying *gain of function* mutants, i.e., mutants that undergo a higher than wild type number of cell divisions before exiting mitosis. We classified 52 mutants as potentially long-lived and manually validated a randomly selected subset of 20. Many of the isolated genes map to biological functions not previously implicated in mitotic senescence, highlighting that the scope of cell processes that impact mitotic longevity is potentially more extensive than currently anticipated.

In order to demonstrate the utility of the isolated genes as relevant genetic portals towards dissecting longevity networks, we undertook a detailed analysis of an ER-Golgi cluster isolated in this screen. Via investigating *RER1*, a prototype member of this cluster, we unraveled a proteostatic network that integrates ER homeostasis into mitotic longevity in yeast. Specifically, we provide evidence that the catabolism of high molecular weight protein aggregates, a natural byproduct of high protein synthesis and turn over in dividing cells, is among the principle drivers of mitotic longevity. We extend these findings by demonstrating that this catabolic network is conserved in design and similarly extends the reproductive lifespan in the nematode *C*. *elegans*.

## Results

### A genome scale screen for mutants with delayed exit from mitosis

During late cell divisions, the yeast *S*. *cerevisiae* display a marked loss of transcriptional silencing at the mating loci [9]. We exploited this hallmark in a pooled collection of 3762 single deletion mutants to search for mutants that undergo a greater than wild type number of cell divisions before exiting mitosis. A full description of the screen design rationale, the isolated set of potential longevity mutants, along with high-resolution validation of a subset of these mutants are outlined in S1, S2, and S3 Figs. Briefly, we integrated the tractable marker orotidin-5'-phosphate decarboxylase *(URA3)* at the *HML* locus in a pool of deletion mutants where non-essential genes were replaced with a *kanMX4* cassette [10]. Cells that undergo loss of silencing at the *HML* locus were selected against using 5-fluoroorotic acid (5-FOA), a cytotoxic uracil analog that inhibits growth of cells expressing *URA3* [11]. Long-lived mutants were predicted to be overrepresented in the pool of dividing cells due to the delayed expression of *URA3* (S1A, S1B, S1C Fig).

**Fig 1:**
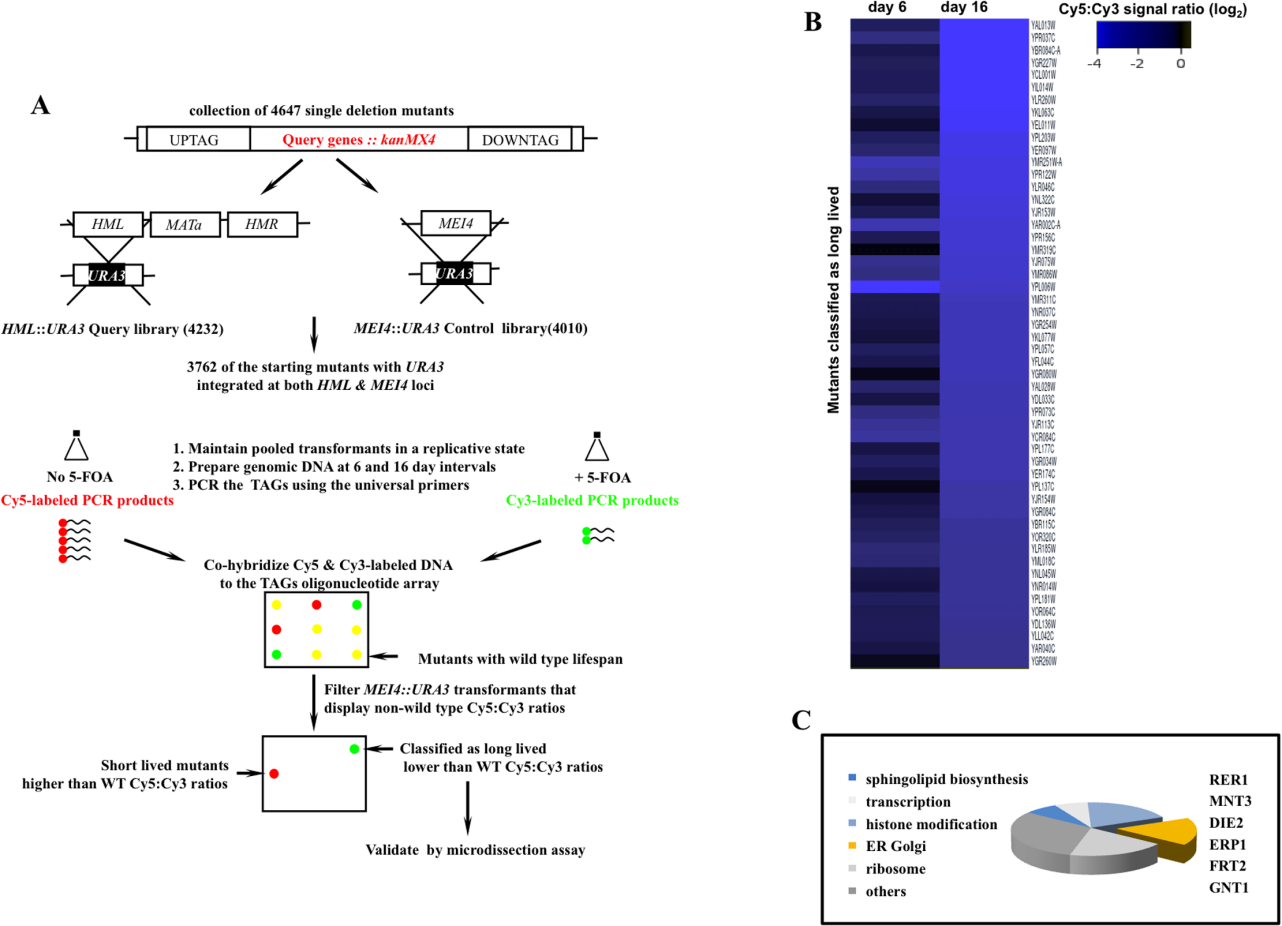
A genome scale screen for isolating mutants with extended mitotic lifespan in the yeast *S*. *cerevisiae*. **A.** The screen work flow. The design rationale is detailed in S1 Fig. **B.** Cy5:Cy3 signal ratios of mutants maintained in a dividing state for 16 days. Ranked values were log_2_ normalized and projected. Of the starting collection of 3762, 52 mutants that maintained negative log_2_ Cy5:Cy3 signal ratios at both day 6 and day 16 and displayed signal ratios <-2.3 at day 16 were classified as potentially long-lived (S3 Fig). **C.** Broad functional clustering of the putative longevity genes isolated in this screen using GO Ontology. Genes that function in protein modification and trafficking across the ER-Golgi network are outlined.

In parallel to the query *HML*::*URA3* library, we also constructed a control library by integrating an identical *URA3* reporter at the meiotically induced *MEI4* locus (*MEI4*::*URA3*). Silencing at both *MEI4* and *HML* loci is mediated via a host of shared gene products [12]. Yet, unlike *HML* locus, *MEI4* remains constitutively silent when cells are maintained in rich growth media. The collective aim of this screen was therefore to isolate mutants that displayed delayed loss of silencing at the *HML* locus while maintaining transcriptional silence at the control *MEI4* locus (Fig 1A).

We screened 3762 deletion strains and classified 52 mutants as potentially long-lived (Figs 1B and S3). This rate of recovery is comparable to a previous screen where 13 mutants were identified as long-lived following a query of 564 mutants using mother daughter dissection [4]. The comparatively modest number of long-lived mutants in these screens may be a reflection of low occurrence frequency of long-lived mutations given the complexity of highly interconnected gene networks that converge on cell division.

A subset of the identified genes mapped to broad cellular processes already associated with mitotic lifespan including protein translation [4], sphingolipid biosynthesis [13], and chromatin remodeling [14,15]. Many of the identified genes however had no previous connection to mitotic longevity. Among these, we recovered a cluster that functions in protein modification and transport across the extensive ER-Golgi network in yeast (Fig 1C). We investigated *RER1*, a prototype member of this gene cluster, in order to understand how the ER-Golgi compartment integrates into longevity networks.

### Inactivating RER1 extends mitotic lifespan in yeast and reproductive lifespan in worms

**Fig 2:**
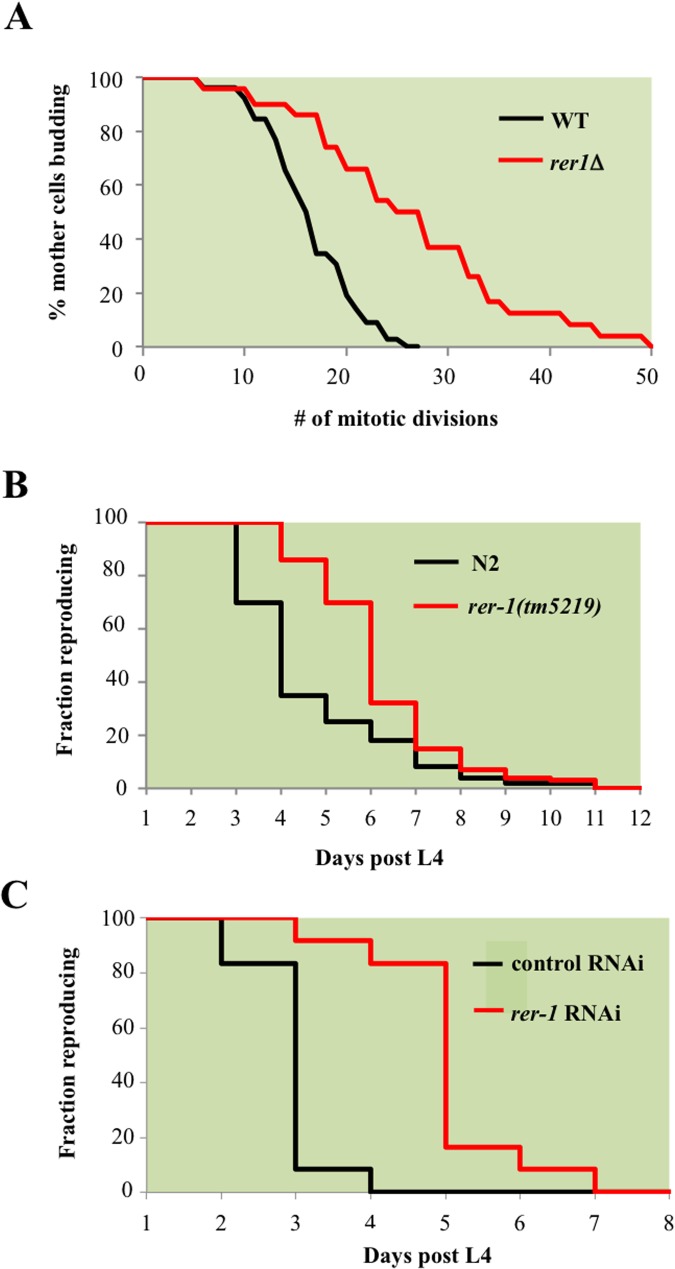
*RER1* inactivation extends mitotic lifespan in yeast and reproductive lifespan in *C*. *elegans*. **A.** Mitotic lifespan of wild type yeast and an isogenic *rer1Δ* grown in complete media (YPD) at 30°C. Buds from a starting population of 40 founding mother cell were successively dissected and counted until all mother cells had ceased dividing. The mean mitotic lifespan for WT and *rer1∆* was 17.5 and 27.5 days, respectively. **B.** Representative reproductive lifespan of self fertilized wild type (n = 85) and *rer-1(tm5129)* (n = 84) hermaphrodites. Fraction of adults generating viable progeny as a function of time is plotted. Day 1 corresponds to the first egg-laying day of adulthood (around 9 hours after the L4 lethargus at 20°C). **C.** Representative reproductive lifespan analysis in worms treated with control (empty vector) or *rer-1* RNAi. In comparison to OP50 supplemented plates **(B),** growth on RNAi plates supplemented with HT115 *E*. *Coli* routinely accelerates the rate of progeny production where on average by the end of day 3 wild type hermaphrodites had generated nearly 90% of their total progeny [48].

*RER1* encodes a receptor that maintains ER compartmentalization by retrieving components of the vesicles that transport cargo from ER to Golgi [16]. Deleting *RER1* increased median lifespan by 40% (p-value < 0.001, nonparametric Mann-Whitney U test), while extending the maximal lifespan by 64% relative to the wild type yeast (Fig 2A).

Rer1 displays extensive sequence homology and functional conservation across eukaryotes (S4 Fig) and human Rer1 functionally replaces its yeast counterpart [17]. We therefore examined whether *rer-1* loss of function impacts reproductive lifespan in *C*. *elegans* by monitoring the ratio of progeny producing animals as a function of time. Hermaphrodite worms exhibit a progressive decline in germ cell proliferation and oocyte fertilization capacity over time that contributes to reproductive cessation. This effect is independent of sperm contribution, brood size, and alterations in reproductive schedule, and instead reflects a physiological deterioration in germ cell quality and/or maintenance that normally occurs as animals age [18]. In *rer-1*(*tm5219*) mutants, harboring a 169 bp deletion of exon 1, reproductive decline was slower compared to the wild type N2 (Fig 2B, p-value < 0.0001, Mantel-Cox test). In late reproductive life (days 6 post L4), when 19% of wild type animals remained reproductive, 51% of *rer-1* hermaphrodites still generated viable progeny (S1A Fig). The prolonged reproductive lifespan in *rer-1(tm5219)* worms was eventually tampered by a sharp increase in cases of matricide, when internal hatching of embryos in actively reproducing old *rer-1* worms led to death (S1B Fig). A comparatively milder RNAi knockdown of *rer-1* with no detectable matricide delayed reproductive senescence in N2 hermaphrodites from day 4 to day 7 of adulthood while increasing the median reproductive lifespan by 66% (Figs 2C and S1D, p-value < 0.0001). Inactivating *RER1* thus extends the window for progeny production in yeast and worms.

### Loss of RER1 induces ER stress in yeast and worms

**Fig 3:**
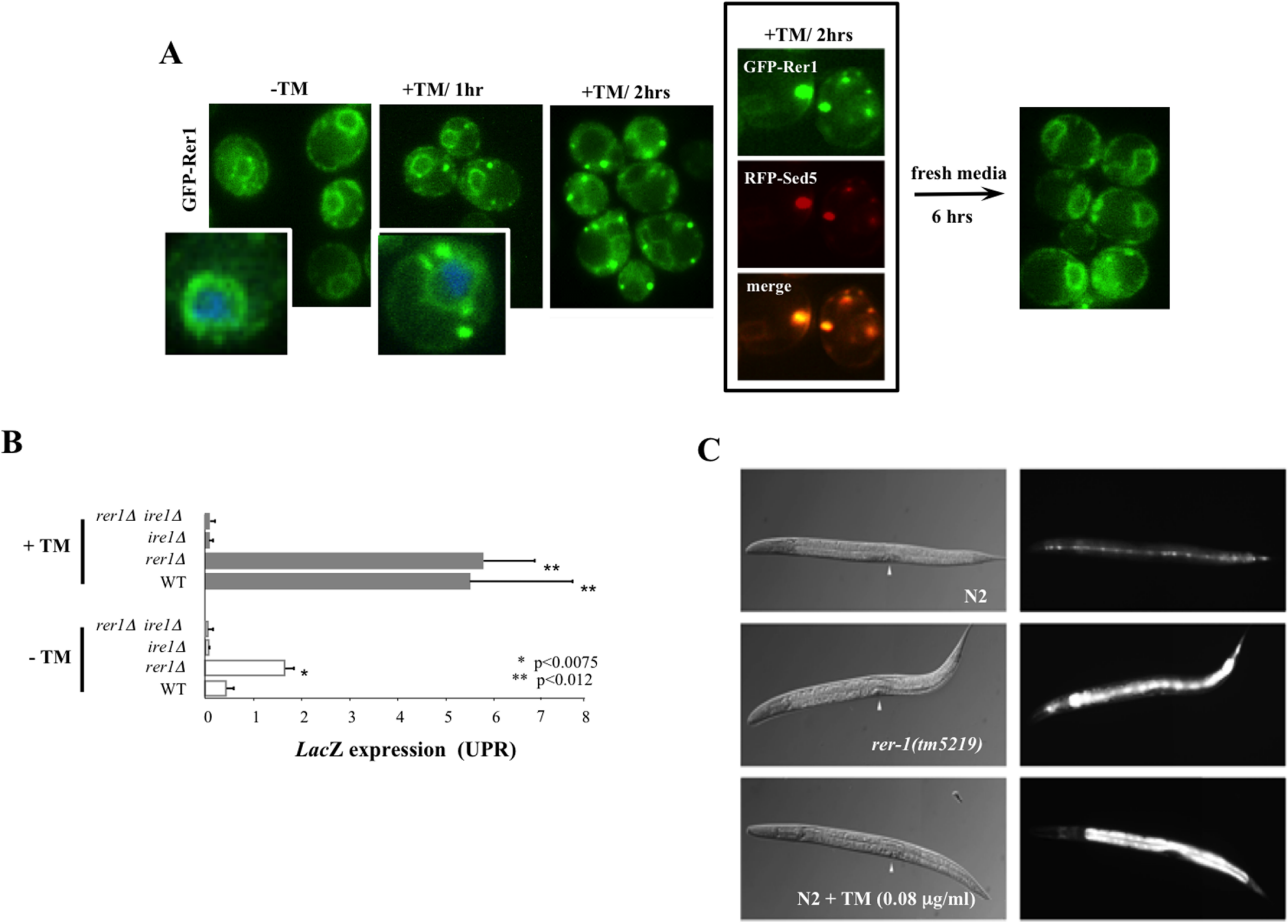
Induction of UPR in yeast and worm *rer1* mutants. **A.** Live cell imaging of GFP-Rer1 expressed from a centromeric plasmid in yeast *rer1Δ* mutants treated with DMSO or after exposure 0.2 μg/ml TM. GFP-Rer1 localizes to perinuclear ER in untreated cells and redistributes to Golgi in cells treated with TM. Insets show cells counterstained with DAPI. Boxed images denote GFP-Rer1 colocalization with Golgi resident RFP-Sed5. GFP-Rer1 relocalizes to ER following a 6 hour release into fresh media. **B.** The *in situ* expression of *LacZ* from a *KAR2* promoter after a 2 hour exposure to DMSO or 0.2 μg/ml TM. Data represent means ± s.e.m. (*n* = 3). **C.** *hsp-4*::*gfp* expression in wild type, *rer-1(tm5219)* or TM-exposed adult hermaphrodites at 20°C. Background fluorescence in untreated N2 is due to basal expression of the GFP reporter in seam cells. Arrowheads denote vulva.

Treatment with ER stressors tunicamycin (TM, Fig 3A) or DTT (S2B Fig), which inhibits protein glycosylation or disulfide bond formation in the ER, respectively, resulted in a marked redistribution of GFP-Rer1 to Golgi. Rer1 relocalization to Golgi was reversible (Fig 3A), not due to a stochastic loss of ER structural integrity (S2C Fig), and further displayed a measure of specificity to ER stress since generic stress including amino acid deprivation or extensive heat shock did not alter GFP-Rer1 *in situ* ER localization (S2D Fig).

In response to ER stress, cells activate an intricate adaptive response, termed the Unfolded Protein Response (UPR). Mediated by a collection of evolutionarily conserved pathways, UPR is aimed at reducing ER protein load in part by signalling a complex program of gene transcription to increase ER protein folding capacity [19]. We hypothesized that the *in situ* redistribution of Rer1 to Golgi reflected a previously unknown component of UPR evoked to reduce ER load via increased trafficking of misfolded proteins from ER to Golgi for their subsequent packaging into autophagosomes destined for proteolysis in the vacuoles [20]. If so, impaired ER to Golgi trafficking in *rer1Δ* mutants should result in elevated ER load. Yeast cells upregulate the transcription of Kar2, a member of the Hsp70 family of molecular chaperones, in response to an increase in ER stress [21]. We monitored *in situ* UPR in *rer1Δ* cells by measuring *lacZ* activity expressed from a *KAR2* promoter. In unperturbed growth (-TM), *lacZ* expression was constitutively elevated in *rer1Δ* mutants relative to the wild type yeast, a reflection of increased ER stress in these cells (Fig 3B). In keeping with its functional conservation, *rer-1* inactivation in worms expressing *hsp-4*::*GFP* [22] led to a uniform increase in expression of the HSP-4 ER chaperone in the intestine (Fig 3C). Constitutively elevated ER stress in *rer1* mutants highlights a requirement for intact ER to Golgi trafficking in maintaining ER homeostasis in yeast and worms.

**Fig 4:**
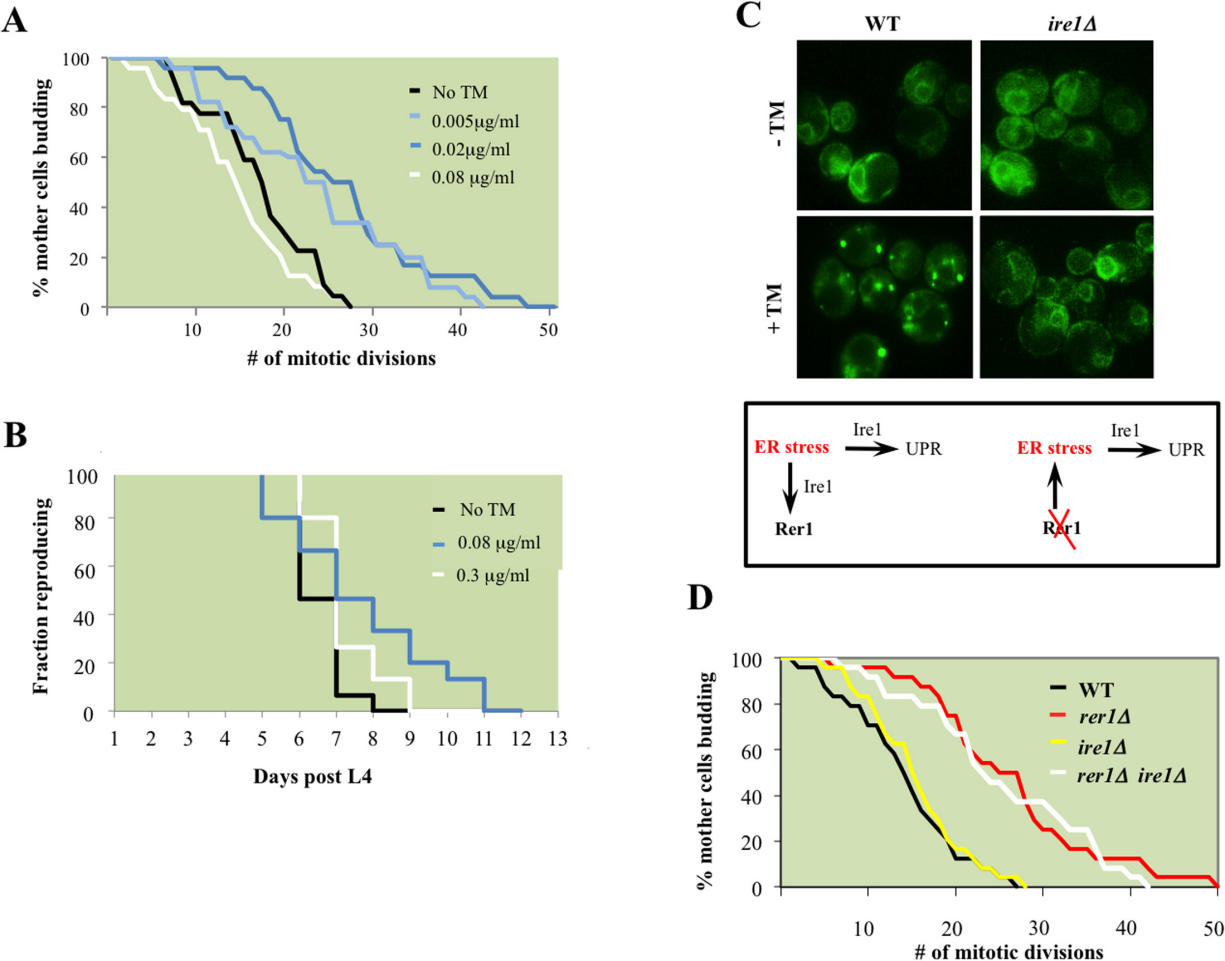
ER stress extends reproductive lifespan in yeast and worms. **A.** Mitotic lifespan of wild type yeast grown on YPD supplemented with the indicated concentrations of TM. Mean mitotic lifespans for WT (18.5) and cells treated with TM at 0.005 (24.5) and 0.02 (27.5) μg/ml were statistically significant (p-values<0.01). **B.** Reproductive lifespan in worms with or without dietary supplementation with TM. **C.** Live cell imaging of GFP-Rer1 in wild type yeast and *ire1Δ* mutants treated with DMSO or after a 2 hr exposure to 0.02 μg/ml TM. (Box) In tandem with inducing canonical UPR, ER stress increases trafficking from ER to Golgi, reflected in the Golgi redistribution of GFP-Rer1 in cells treated with TM. Conversely, deleting *RER1* increases ER load denoted by the high basal UPR in *rer1Δ* mutants. **D**. Mitotic lifespan of WT (16.5 days) and isogenic *rer1∆* (26.5), *ire1∆* (16.5) and *rer1∆ ire1∆* (24.5) mutants at 30°C. Differences between WT mean mitotic lifespan were significant for *rer1∆* and *rer1∆ ire1∆* mutants (p-values<0.001).

We next examined whether high basal ER stress drives mitotic longevity. To this end, we monitored lifespan in wild type yeast cells chronically exposed to TM. Growth in 0.02 μg/ml TM, 100x less than the concentration refractory to growth (S3 Fig), extended median lifespan in yeast (Fig 4A). Remarkably, TM also delayed reproductive senescence in wild type *C*. *elegans* (Fig 4B). Our data suggest that exposure to chronic sublethal ER stress can autonomously signal reproductive longevity in both yeast and worms.

### ER stress extends lifespan in an Ire1-independent manner

The principle sensor of UPR is encoded by *IRE1* in yeast and its conserved orthologs in higher eukaryotes [23]. The ER membrane protein Ire1 initiates UPR in response to accumulation of misfolded proteins in the ER lumen. Relative to wild type cells, *ire1Δ* mutants displayed constitutively impaired basal UPR (Fig 3B), failed to activate UPR after TM treatment (Fig 3B), and as a consequence were exquisitely sensitive to TM (S3 Fig). Our analyses suggest that *rer1∆* and *ire1∆* genetic interactions are highly complex. First, Ire1 signaled the redistribution of Rer1 to Golgi after TM treatment as GFP-Rer1 maintained an ER localization in *ire1Δ* mutants treated with TM (Fig 4C). Second, deleting *IRE1* entirely abolished the otherwise elevated basal UPR in *rer1Δ* mutants, indicating a requirement for sensing protein load in the ER lumen by Ire1 (Fig 3B). Finally, deletion of *RER1* did not alter *ire1∆* TM sensitivity (S3 Fig). Collectively these data suggest that trafficking across the ER-Golgi network is functionally coupled to protein load in the ER lumen. In response to an increase in ER load, Ire1 signals an increase in cargo transport from ER to Golgi, a critical intermediary step in removing misfolded proteins from ER lumen since Golgi, but not ER, is the principle donor of membrane bilayers required for expansion of autophagosomes which ultimately traffic protein cargo imported from ER to vacuoles for degradation [20,24]. In cells with elevated ER load, Rer1 is preferentially distributed to Golgi, presumably because it is required for the retrieval of transport vesicles back to the ER for subsequent rounds of cargo transport [16]. Conversely, deleting *RER1* short circuits this pathway, leading to increased protein load in the ER and the ensuing activation of UPR (Fig 4C, lower panel).

Surprisingly, despite its constitutive induction, Ire1-mediated canonical UPR does not contribute to mitotic longevity in yeast; in line with a previous report [4], *ire1Δ* mutants had a wild type lifespan despite a highly impaired UPR (Fig 4D). Moreover, deleting *IRE1* did not impact the otherwise extended lifespan in *rer1Δ*. While integral to survival under high ER stress, the Ire1-mediated UPR is dispensable in proliferative growth (S3 Fig) and mitotic longevity in yeast (discussed below).

### Induction of autophagy in yeast and worm rer1 mutants

**Fig 5:**
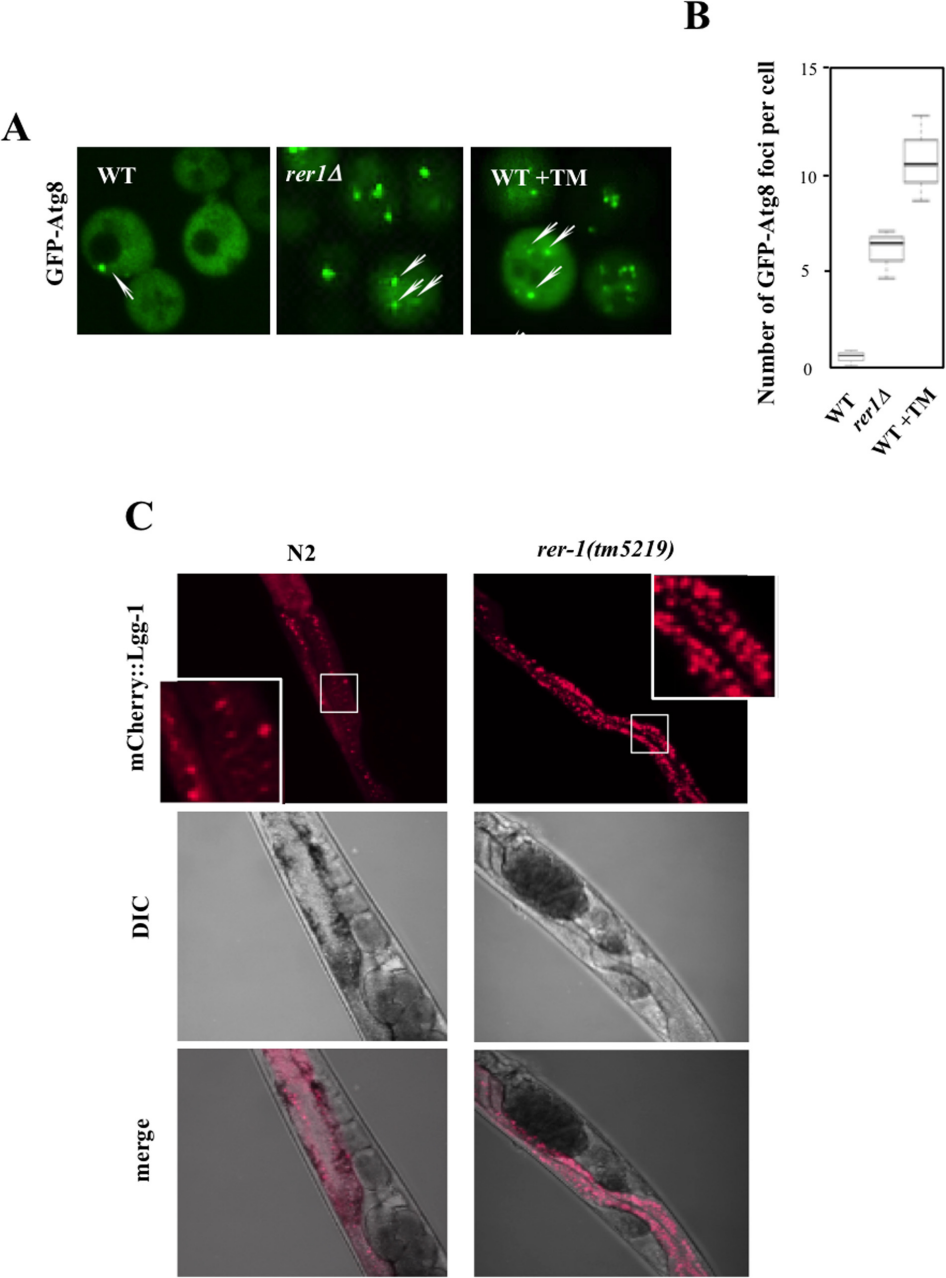
Induction of autophagy in yeast and worm *rer1* mutants. **A.** Live cell imaging of GFP-Atg8 foci (arrows) in wild type cells after a 2 hr exposure to TM (0.02 μg/ml) and *rer1Δ* cells. **B.** Microscopically detectable GFP foci in individual yeast cells were scored from digitized Z-stacked images by an observer blind to the genotype. Data represent means ± s.e.m. (n>20). **C.** Intestinal expression of mCherry-LGG-1 (*P*_*nhx-2*_ *mCherry*::*lgg- 1*) in day2 wild type and *rer-1(tm5219)* hermaphrodites. Insets represent magnification of the boxed sections.

In tandem with inducing UPR, ER stress signals the activation of (macro) autophagy in yeast [25] and human cells [26]. Autophagy is a conserved housekeeping mechanism whereby bulk cytoplasm, including proteins and organelles, are sequestered into membrane-bound vesicles (autophagosomes) which subsequently fuse with vacuoles where their content is degraded by a complement of vacuolar proteases [27]. While mechanistically distinct, UPR and autophagy synergistically mitigate ER protein load. We therefore monitored autophagy in yeast *rer1Δ* cells by using the *in situ* reporter GFP-Atg8, a functional allele of *ATG8* encoding an autophagosome assembly factor [28]. In wild type cells, GFP-Atg8 had a diffuse cytoplasmic distribution with sporadic foci, a reflection of low basal autophagy in unperturbed growth (Fig 5A). In keeping with their elevated ER load, untreated *rer1Δ* cells or wild type cells treated with a life-extending dose of TM (0.02 μg/ml) routinely had multiple GFP-Atg8 foci (quantified in Fig 5B), denoting increased number of assembled autophagosomes in the cytoplasm.

Exposure to high concentrations of TM induces a selective form of microautophagy, termed ER-phagy, where ER is taken up by vacuoles via invagination of the vacuolar membrane, presumably to reduce the expanded ER volume in these cells [29]. We found that exposure to 1 μg/ml TM led to robust cleavage of Sec63-GFP, a proxy readout for vacuolar internalization of ER [30]. However exposure to 0.02 μg/ml TM, a dose that extends lifespan, did not result in a detectable induction of microautohagy even after prolonged exposure (S4 Fig). Extension of lifespan following chronic treatment with sublethal concentrations of TM therefore does not appear to be accompanied with induction of microautophagy.

We similarly monitored (macro) autophagic flux in the intestine of adult worms expressing an mCherry fusion of LGG-1, the *C*. *elegans* Atg8 ortholog *(Pnhx-2*::*mCherry*::*lgg-1*) [31,32]. In wild type worms, mCherry::LGG-1 exhibited a diffuse signal with sporadic punctate foci formation (Fig 5C). In *rer-1(tm5219)* worms however the number of foci was significantly increased, highlighting a higher abundance of autophagosomes. Elevated ER stress in *rer1* mutants therefore signals robust formation of autophagosomes in both yeast and worms.

### Protein aggregates are cleared with increased efficiency in yeast and worm rer1 mutants

Autophagy contributes to cell homeostasis in part by removing cytoplasmic protein inclusions [27]. We asked whether the high basal autophagy in *rer1Δ* mutants culminated in a more efficient clearance of protein aggregates from the cytoplasm. We analyzed the content of the detergent insoluble protein inclusions in yeast *rer1Δ* and its wild type counterpart by SDS-PAGE [33]. Consistent with their expanded pool of autophagosomes, *rer1Δ* mutants or wild type cells treated with TM had reduced levels of detergent insoluble protein inclusions relative to the wild type cells (S5 Fig).

To obtain direct evidence for the apparent reduction in protein aggregates in *rer1Δ*, we monitored the *in situ* steady state of human α-synuclein-GFP (α-syn-GFP) expressed in yeast [34]. Formation of α-syn aggregates in the brain temporally coincides with the onset of a host of human neurodegenerative disorders broadly associated with aging [35].

**Fig 6:**
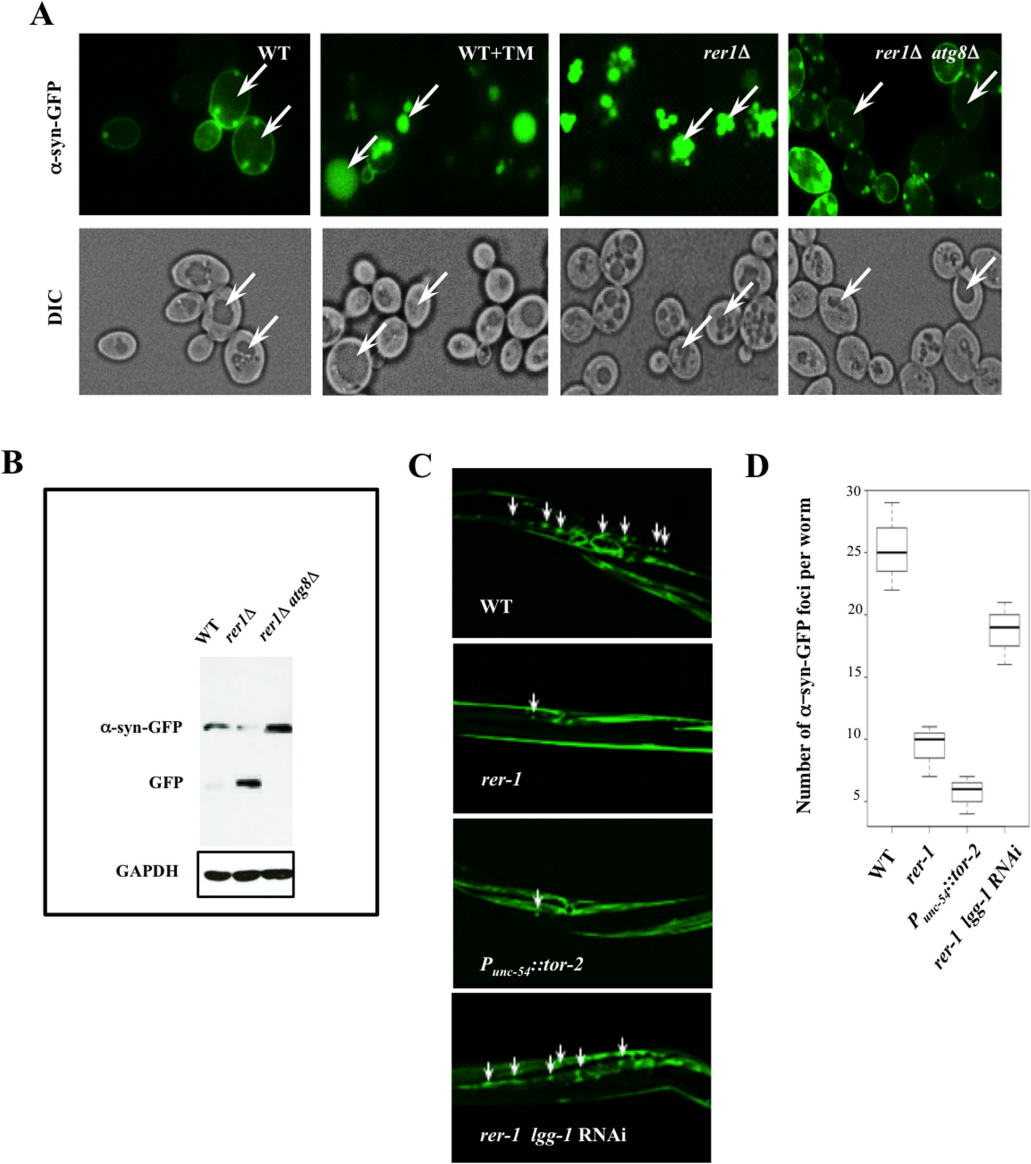
Enhanced clearance of protein aggregates in yeast and worm *rer1* mutants. **A.** Live cell imaging of α-syn-GFP after a 4 hr induction in 0.2% galactose. TM (0.02 μg/ml) was added to the cultures after an initial induction in galactose and cells were imaged 2 hours later. In a marked contrast to untreated cells, the GFP foci were largely vacuolar in *rer1Δ* or wild type cells treated with TM (arrows). **B.** GFP immunoblot in whole cell lysates prepared from cells in (**A)**. The intravacuolar proteolysis of α-syn-GFP generates the transiently stable GFP moiety detected as a discrete fragment in SDS-PAGE. **C.** Fluorescent images of transgenic hermaphrodites stably expressing α-syn-GFP from a body wall muscle promoter (*Punc-*_*54*_::*α-syn*::*GFP*). Arrows denote a subset of the α-syn-GFP foci. Worms co-expressing heat shock protein torsinA (*tor-2*) in the same cells (*P*_*unc-54*_::*tor-2*) served as a positive experimental control. The average number of α-syn-GFP foci per worm scored from digitized Z-stacked images ± s.e.m are plotted in **D** (n >20).

When expressed in wild type yeast, human α-syn-GFP forms prominent cytoplasmic foci (Fig 6A). In striking contrast to wild type cells, GFP foci in *rer1Δ* or wild type cells treated with TM displayed a predominantly vacuolar localization with the cytoplasm essentially devoid of microscopically detectable foci (Fig 6A), a profile consistent with the reduced level of detergent insoluble protein inclusions in these cells (S5 Fig). The apparent clearance of aggregates in yeast *rer1Δ* was due to the constitutively high basal autophagy in these cells since deleting *ATG8* restored α-syn-GFP cytoplasmic foci and concomitantly abolished its intravacuolar proteolysis (Fig 6A and 6B).

Ectopic expression of human α-syn-GFP gives rise to microscopic foci in body wall muscle cells of adult worms stably expressing *Punc-*_*54*_::*α-syn*::*GFP* (Fig 6C) [36]. Consistent with their high basal autophagy, *rer-1(tm5219)* worms or a positive experimental control over- expressing heat shock protein torsinA (*tor-2*) (*P*_*unc-54*_::*tor-2*) [37], displayed a marked reduction in the number of the GFP foci (Fig 6C, quantified in 6D). An RNAi knockdown of *lgg-1* largely restored the α-syn-GFP foci in muscle cells of *rer-1* animals, highlighting a requirement for intact autophagy in removal of protein aggregates in worms. Collectively, these data demonstrate that the expansion in the pool of autophagosomes in *rer1* mutants is accompanied with a quantitative reduction in cytoplasmic protein inclusions in yeast and worm.

### Autophagy is required for extended lifespan in yeast and worm rer1 mutants

**Fig 7:**
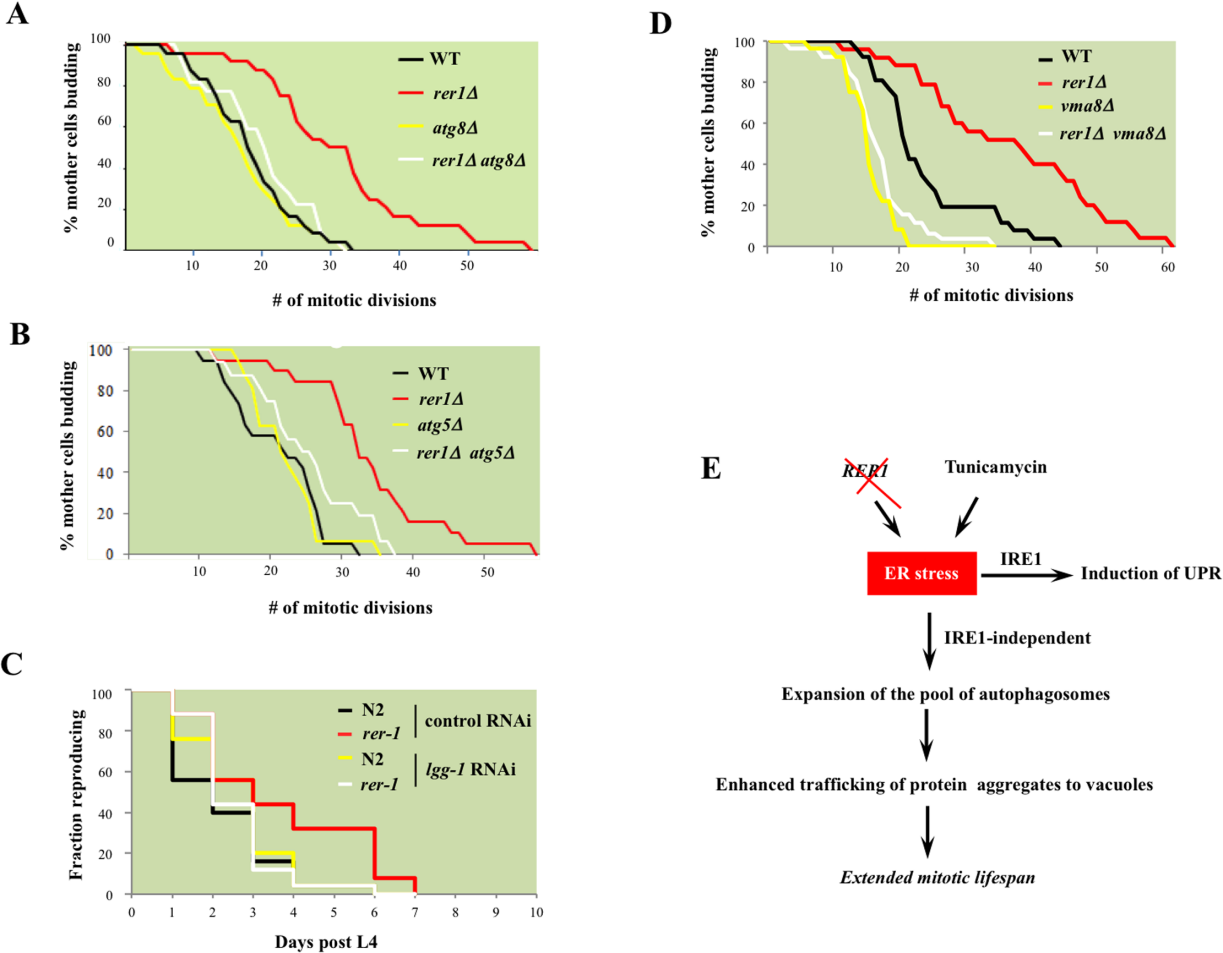
The requirement for intact cellular catabolism in extension of lifespan in yeast and worm *rer1* mutants. Mitotic lifespan in wild type or *rer1Δ* mutants harboring a deletion of the autophagosome components *ATG8* **(A)** or *ATG5* **(B).** The mean mitotic lifespans for the samples in **(A)** were WT (16.5 days), *rer1∆* (27.5), *atg8∆* (15.5) and *rer1∆ atg8∆* (18.5). The *rer1∆* mean mitotic lifespan was significantly different from the WT (p-value<0.01), whereas the differences between WT, *atg8∆* and *rer1∆ atg8∆* were not. Similarly, in **(B)** the mean mitotic lifespan difference between WT (17.5 days) and *rer1∆* (38.5) was statistically significant (p-value<0.01), whereas the differences between WT, *atg5∆* (17.5), and *rer1∆ atg5∆* (18.5) were not. **C.** Reproductive lifespan in N2 (n = 25) and *rer-1(tm5219)* (n = 25) worms treated with control or *lgg-1* RNAi. **D**. Deletion of *VMA8*, encoding a vacuolar proton pump subunit, abolishes the extended lifespan in yeast *rer1Δ* mutants. The differences between WT (21.5 days), *vma8∆* (15.5; p-value < 0.01) and *rer1∆ vma8∆* (17.5; p-value < 0.05) were statistically significant. **E.** Autophagy, induced in response to elevated ER stress in *rer1Δ* or following exposure to the ER stressor (TM), traffics cytoplasmic aggregates to the vacuoles and in doing so delays mitotic senescence (details in text).

Next we asked whether the constitutively high basal autophagy contributes to mitotic longevity in *rer1* mutants. Deletion of genes encoding the autophagosome components Atg8 or Atg5, which mediates Atg8 lipidation required for expansion of autophagosomes [28], abolished the otherwise extended mitotic lifespan in yeast *rer1Δ* (Fig 7A and 7B). Knockdown of *lgg-1* similarly reduced the reproductive lifespan in *rer-1* worms (Fig 7C). Enhanced proteostasis thus appears to underlie the extended lifespan in yeast and worm *rer1* mutants.

To examine this observation further, we monitored mitotic lifespan in yeast with functionally impaired vacuoles. Vacuoles constitute the biological endpoint for catabolism of protein aggregates. The vacuolar acidic milieu, required for the optimal enzymatic activity of its ensemble of hydrolases, is maintained by a proton-coupled ATPase complex [38]. Deletion of *VMA8*, encoding a component of the vacuolar ATPase, reduced mitotic lifespan in wild type yeast and concomitantly abolished the extended lifespan in *rer1Δ* mutants (p-value < 0.01, Fig 7D). The strict requirement for both intact autophagy and vacuolar proteolysis points to enhanced catabolic output as the principle driver of longevity in yeast *rer1Δ* mutants.

In our screen we also recovered *MNT3*, encoding a Golgi glycosylase, among the long-lived ER-Golgi cluster of mutants (Fig 1C). As in *rer1Δ* mutants, deletion of *MNT3* led to high basal UPR, elevated autophagy, and extended mitotic lifespan (S10A, S10B, and S10C Fig). Moreover, deleting *MNT3* did not further enhance the mitotic lifespan in *rer1∆* cells, indicating that both mutants converge on autophagy as the longevity mechanism (S10C Fig).

## Discussion

### A genome scale screen for identification of mutants that undergo an atypically high number of mitotic divisions before exiting mitosis

Here we report a screen for identification of yeast mutants that undergo a higher than wild type number of cell divisions before exiting mitosis. We classified 52 mutants as potentially long-lived. In a high resolution mother-daughter assay of a randomly selected subset of 20 mutants in this panel, 7 displayed a >15% increase in mean mitotic lifespan relative to the wild type, validating the relevance of this screen as a gene enrichment platform.

Many of the putative longevity genes isolated in this screen map to biological processes with no previous annotation to mitotic lifespan. These include the cAMP-dependent kinase catalytic subunit (*TPK2*), the regulatory subunits of protein phosphatase 1 (*GLC8* and *GIP3*) and phosphotyrosine *LTP1*, the polyamine transporter (*TPO3*), tRNA thiouridylase (*SLM3*), and the Glutathione-dependent oxidoreductase (*GRX4*). The potential involvement of these processes in extending mitotic longevity indicates that the regulatory scope of mitotic lifespan may be broader than currently anticipated. Of the 52 potential longevity genes isolated in this screen, 32 have functional orthologs in humans (S3 Fig). This gene set should serve as a starting genetic portal in mapping mitotic longevity pathways in human cells.

### Functional coupling of ER homeostasis to mitotic lifespan in yeast and worms

We recovered a gene cluster that functions in ER to Golgi protein transport (*RER1*, *ERP1*) and N-linked protein glycosylation (*DIE2*, *MNT3*, *GNT1*). We focused on *RER1* to understand how ER integrates into mitotic longevity. Localized to perinuclear ER in unperturbed growth, Rer1 redistributes to Golgi after treatment with ER stressors (Figs 3A and S2B). Redistribution of Rer1 to Golgi is reversible, specific to ER stress, and requires Ire1, the principle inducer of UPR. These observations suggest that Rer1 functions downstream of ER stress and in synergy with canonical UPR where increased ER to Golgi trafficking likely contributes to ER homeostasis by removing misfolded proteins from the ER lumen to Golgi for their subsequent transport to vacuoles by autophagosomes.

Despite its constitutive induction, canonical Ire1-mediated UPR does not contribute to mitotic longevity in yeast *rer1∆* mutants; *ire1∆* mutants display wild type lifespan despite a complete absence of UPR. Moreover, deletion of *IRE1* does not impact the extended mitotic lifespan in *rer1Δ* mutants. While integral to survival under ER stress, Ire1 is dispensable for growth (S3 Fig) and mitotic longevity in yeast (Fig 4D). To our knowledge a contribution of Ire1-mediated UPR to mitotic lifespan in other eukaryotic models has not been examined.

### Enhanced protein catabolism delays mitotic exit in yeast and reproductive senescence in worms

In tandem with UPR, yeast *rer1Δ* mutants activate autophagy, likely to mitigate an increase in ER load. While complementary in function, we show that induction of autophagy and UPR can be mechanistically uncoupled; despite a strict requirement for Ire1 in initiating canonical UPR (Fig 3B), Ire1 is dispensable for inducing autophagy in yeast (S6 Fig). Our data are in line with a previous report indicating that sensors other than Ire1 signal autophagy in response to increased ER load [29].

We have provided evidence that the elevated basal autophagy in response to high ER stress underlies the extended lifespan in yeast and worm *rer1* mutants (Fig 7A, 7B, and 7C). Deletion of *MNT3* similarly leads to high basal ER stress, the ensuing increase in the pool of autophagosomes, and extended mitotic longevity (S10 Fig). The induction of autophagy thus appears to be a broad adaptive response evoked to augment UPR in cells with impaired protein modification (*mnt3Δ)* or trafficking (*rer1Δ*) across the ER-Golgi network.

The induction of autophagy in *rer1Δ* mutants is accompanied by a marked clearance of α-syn cytoplasmic inclusions, an observation consistent with the role of autophagy as the principle pathway for removing high molecular weight protein aggregates (Fig 6A and 6C). Protein aggregates form prominent cytological features that segregate with and contribute to senescence in post mitotic models including *C*.*elegans* and mice [39,40]. Our data suggest that as in post mitotic aging, formation of protein aggregates also serves as a determinant of reproductive senescence in yeast and worms (Fig 7E).

Finally, we note that despite their extended lifespan, yeast *rer1Δ* mutants remain sensitive to ER stressors (S3 Fig), display elevated loss of viability after DNA damage [41], and grow poorly under low nutrient condition [42]. While autophagy extends the duration of reproduction, it does not ameliorate other phenotypic defects in *rer1* mutants. From an evolutionary viewpoint, the targeted induction of autophagy in *rer1* mutants provides a compelling example of a Darwinian adaptation aimed at extending the window for offspring production.

## Materials and Methods

### Yeast strains and molecular biology

Unless indicated otherwise, experiments were carried out in BY4741 (*MAT* **a** *his3Δ1 Δleu2 Δmet15 Δura3*). Respective null mutants were constructed by single step PCR-based gene deletion. Strains were grown in YPD or SC media supplemented with 30 μg/ml of all amino acids. For inducing nutritional stress, cells were grown in SC media without amino acids. Throughout, cells expressing GFP-Atg8 were grown in SC-ura media.

### Yeast methods

A *CEN* based plasmid containing a *lacZ* gene expressed from a *KAR2* promoter [21] provided an *in situ* readout for UPR. Yeast vacuolar enzymatic activity was measured as described [43]. Total yeast protein lysates were prepared by bead beating as described [44]. Mouse anti-GFP monoclonal antibody (Covance) was used at 1/5000.

### Yeast mitotic lifespan analysis

Lifespan analyses were essentially as described [45]. Briefly, cells from logarithmically growing liquid cultures were plated on YPD. After an overnight incubation at 30°C, a minimum starting population of 40 founding daughter cells were removed with a Zeiss Micro-manipulator and buds were successively dissected until all mother cells had ceased dividing. To monitor the effect of ER stress on longevity, tunicamycin (Sigma Aldrich) or DMSO was added to the plate mix. Each experiment was performed at least twice, with most repeated 3 to 5 times. Significance of the differences in mean mitotic lifespan for each experiment was determined using the nonparametric Mann-Whitney U test, as done previously [46]. Eight data points flanking the 50% mitotic lifespan point for each experiment were used in the Mann-Whitney U-value calculator (http://www.socscistatistics.com/tests/mannwhitney/Default.aspx).

### Analysis of worm reproductive lifespan

With the exception of RNAi experiments which used an *rrf-3(pk1426)* temperature sensitive background grown at 16°C, all other experiments were performed at 20°C. Embryos were isolated by hypochlorite treatment and L1 synchronized in diapause. Larvae were allowed to develop to late L4 stage (44 hrs at 20°C or 57 hrs post embryonic development at 16°C) when they were singled out on NGM plates. The reproductive capacity was determined by scoring the number of viable progeny daily. The end of reproductive lifespan was assigned as the last day with progeny followed by two consecutive days without progeny production. Sterile hermaphrodites were excluded from the data. When indicated (Fig 4B), tunicamycin was added to the UV irradiated bacteria (OP50) food supplement. The Mantel-Cox test was used to calculate P-values in the different reproductive lifespan curves.

### Monitoring autophagy in *C*. *elegans*

Expression of LGG-1 was monitored at 20°C in adult transgenic worms carrying an integrated *P*_*nhx-2*_::*mCherry*::*lgg-1* transgene. Age-matched wild type and *rer-1(tm5219)* animals were grown and imaged in parallel.

### Analysis of protein aggregates

We used a published protocol [47] to monitor the formation of detergent insoluble materials in yeast. Briefly, spheroplasts were prepared and lysed in a buffer containing 1% sarkosyl and incubated on ice for 5 minutes with occasional vortexing. 2 mg of each lysate was precleared with a 10 minute spin at 1,000g and the detergent insoluble material were pelleted by a 1 hr spin at 20,000g at 4°C. Pellets were resolubilized in 8M urea containing 2% TX-100, resolved by SDS-PAGE, and analyzed by coomassie blue staining. α-syn-GFP expression was induced by growing cells in 0.2% galactose for 4 hours.

### Microscopy

Log phase yeast culture cells were spotted on a glass slide and imaged with an Olympus BX-51 microscope equipped with Infinity software v.5.0.3 (Lumenera, Ottawa, Canada) for image acquisition. Alternatively, live cells were imaged using a laser scanning confocal microscope (Zeiss LSM410). MetaMorph v6.1 software (Universal Imaging Corporation; Downington, PA) was used to construct calibrated overlays of z-stacked images. Worms were mounted on 2% agarose pads spotted with 20 μl of 10 mM Na-azide. Confocal microscopy was performed using a Zeiss LSM410 confocal laser scanning microscope. Z-stacks were acquired and processed using the Zeiss LSM software package. Alternatively, imaging was performed using an Applied Precision DeltaVision microscope (GE). Image processing was performed using Softworx software (G.E./Applied Precision).

## Supporting Information

S1 Fig A. The screen design rationale. In yeast undergoing early mitotic divisions, the two repositories of the mating type information, *HMR* and *HML*, are maintained in a heterochromatic state and are transcriptionally silent [49]. In late mitotic divisions, progressive loss of heterochromtic state leads to loss of transcriptional silencing at the mating loci. We replaced the HML locus in a pool of yeast single deletion mutants with the tractable marker orotidin-5'-phosphate decarboxylase (*URA3*). Cells that display loss of silencing at the *HML* locus are selected against using 5-fluoroorotic acid (5-FOA), a cytotoxic uracil analog that inhibits growth of cells expressing *URA3*. The age-dependent derepression of the mating loci occurs at random during the latter cell divisions (arrows), typically in cells that have completed between 70 and 100 percent of their mitotic divisions [9]. For instance wild type yeast with an average lifespan of 30 generations give rise to a population of 2^30^ daughter cells in the absence of 5-FOA, whereas in the media containing 5-FOA they randomly generate 2^21^−2^30^ daughters. Importantly, the onset of *URA3* expression is governed by the mitotic lifespan intrinsic to each mutant; *HML*::*URA3* mutants with short mitotic lifespan are preferentially depleted from the pool of mutants in the presence of 5-FOA due to an earlier expression of *URA3*. In contrast mutants with prolonged mitotic lifespan are overrepresented due to the delayed expression of *URA3*. The screen schematic is outlined in Fig 1A. We started with a collection of haploid deletion strains of 4647 nonessential genes [10]. The *kanMX4* deletion cassettes are flanked by UPTAG and DOWNTAG sequences unique to each deleted locus. We replaced the *HML* locus in the pool of mutants with a *URA3* reporter via a one-step integration by homologous recombination. In parallel to the query *HML*::*URA3* library, we also constructed a control library by integrating an identical *URA3* reporter at the meiotically induced *MEI4* locus (*MEI4*::*URA3*). Silencing at both *MEI4* and *HML* loci is mediated via a host of shared gene products including histones and chromatin assembly factors [12,50]. Yet unlike the *HML* locus, the *MEI4* locus remains constitutively silent when cells are maintained in rich growth media [51]. The collective aim of this screen was therefore to isolate mutants that display delayed loss of silencing at the *HML* locus while maintaining transcriptional silence at the control *MEI4* locus. Of the starting collection of 4647 mutants, we recovered 4232 *HML*::*URA3* and 4010 *MEI4*::*URA3* transformants. The high transformation efficiency was largely due to the extensive 1kb sequence homology between the integrative *URA3* fragments and the insertion loci. The remainder of the mutants did not form URA+ colonies presumably because they were not transformed or had basal defects in *URA3* expression. 3762 of the single deletion mutants harbored selectable *URA3* markers at both *HML* and the control *MEI4* loci. These mutants, encompassing 79% of all non-essential genes in yeast, were analyzed further. We grew the query *HML*::*URA3* and control *MEI4*::*URA3* libraries in media supplemented with or without 5-fluoroorotic acid (5-FOA). We maintained the pool of mutants in a continually dividing state by repeated seeding into fresh YPD media, prepared genomic DNA at 6 and 16 day intervals, and amplified the kan cassettes by PCR using universal primers [10]. PCR products from the cells grown in media supplemented with or without 5-FOA were labeled with Cy3 and Cy5, respectively, and cohybridized to a high-density array of the TAG sequences. All subsequent steps including hybridization, and image capture and analysis were conducted essentially as described [10,52]. The Cy5:Cy3 signal ratio of each unique TAG reflected the relative abundance of the corresponding mutant at the sampling intervals. We similarly profiled the abundance of *MEI4*::*URA3* mutants grown mitotically for 16 days. This important experimental control allowed for identification of false positives with stochastic defects in *URA3* expression, for instance those with constitutive defects in chromatin assembly, gene expression, or uracil biosynthesis pathways. Of the starting collection of 3762, 52 mutants that maintained negative log_2_ Cy5:Cy3 signal ratios at both day 6 and 16 and displayed a signal ratio of < -2.3 at day 16 were classified as long lived. **B.** Growth profile of previously annotated longevity mutants harboring *URA3* at the *HML* locus (*HML*::*URA3*). Fivefold serial dilutions of cells were spotted on plates and colonies were imaged after a 2 day growth at 30°C. All mutants displayed comparable growth in the absence of 5-FOA (Ura-). The prolonged maintenance of silencing the *HML* locus resulted in delayed 5-FOA cytotoxicity in long-lived mutants (*fob1Δ*, *gpa2Δ)*. In contrast, earlier onset of *URA3* expression conferred enhanced 5-FOA cytotoxicity in a short-lived mutant *(lag2Δ)*. A long-lived mutant isolated in this screen, *rer1Δ*, is included for comparison. **C.** A small-scale validation of previously annotated longevity mutants as a proof of principle for the screen. We maintained the pool of mutant in a mitotic state in media supplemented with or without 5-FOA, prepared genomic DNA after16 days, and PCR amplified the kan cassettes using gene specific primers. All mutants were comparably represented when harboring *MEI4*::*URA3* or when grown in media without 5-FOA. In the presence of 5-FOA, however, short lived *lag2Δ* mutant was preferentially depleted from the *HML*::*URA3* pool while the *gpa2Δ* long-lived mutant was over- represented. **D.** The recovery rate of previously annotated long-lived mutants. Of the 81 long-lived single gene deletion mutants curated in SGD (www.yeastgenome.org), 62 harbored the *URA3* reporter at both *HML* and the control *MEI4* loci and were analyzed in our screen. Of these 62 long-lived mutants, 19 were correctly classified as long-lived as they maintained negative log2 Cy5:Cy3 signal ratios throughout and displayed a signal ratio of < -2.3 at day 16. The correctly classified longevity mutants are denoted by asterisks in the projected log2 Cy5:Cy3 ratios.
(TIFF)

S2 Fig Mitotic lifespan for a randomly selected subset of 20 mutants isolated in this screen. A minimum of 40 mother cells for each strain grown on YPD media at 30°C were assayed by mother-daughter microdissection. Values denote percent change in mean mitotic lifespan relative to the parental wild type assayed in parallel.
(TIFF)

S3 Fig Mutants classified as potentially long lived and their respective log2 Cy5:Cy3 signal ratios at day 6 and day 16. Human functional orthologs are shown.
(TIFF)

S4 Fig Rer1 protein sequence conservation in eukaryotes. Yeast Rer1 has 51% amino acid identity to human and 48% identity to its *C*. *elegans* orthologs.
(TIFF)

S5 Fig A. Reproductive lifespan analysis in self fertilized wild type and *rer-1(tm5219)* hermaphrodites. The fraction of reproducing animals was calculated by determining the proportion of adults generating progeny as a function of time. Day 1 corresponds to the first egg-laying day of adulthood (around 9 hours after the L4 lethargus at 20°C). **B.** Cumulative percentage of matricide in wild type and *rer-1(tm5219)* mutants. Matricide or “bagging” occurs when fertilized oocytes are retained in the uterus where the embryo and larva ultimately develop. In wild type worms, matricide generally occurs in late stages of reproductive life due to the progressive deterioration of somatic tissues that support egg laying. Unlike wild type worms, matricide levels in *rer-1* mutants sharply increased from day 5 onwards, indicating a disconnect between germline and somatic aging. As matricide removes reproductively active animals from the population, it leads to an underestimation of the reproductive lifespan as well as the number of progeny generated in *rer-1* mutants. *rer-1* expression (**C)** and reproductive lifespan **(D)** in wild type hermaphrodites treated with control (empty vector) or *rer-1* RNAi. Experiments were performed at 16°C using the *rrf-3(pk1426)* sensitized background.
(TIFF)

S6 Fig Golgi redistribution of GFP-Rer1 after ER stress. **A.** Growth of *rer1Δ* carrying a *CEN* plasmid expressing an N-terminal GFP fusion of Rer1 (GFP-Rer1) or GFP alone with or without TM. Fivefold serial dilutions of yeast cells were spotted and colonies imaged after a 2 day growth at 30°C. GFP-Rer1, but not GFP alone, restored growth in *rer1Δ* mutants in the presence of TM. **B.** GFP-Rer1 relocalization to Golgi after a 2hr treatment with 2mM of the ER stressor DTT. **C.** Fluorescent images of the GFP fusion of ER proteins Sec71 and Sec12 in normal growth and after a 4 hour exposure to TM. **D.** Live cell fluorescent images of GFP-Rer1 in cells treated with TM for 2 hours, grown in SC media without amino acids (-aa) or heat shocked at 37°C for 6 hours. GFP-Rer1maintained ER localization in all but TM treated cells.
(TIFF)

S7 Fig The growth profile of *rer1Δ* mutants in the presence or absence of TM. Fivefold serial dilutions of cells were spotted on plates containing the indicated amounts of TM. Colonies were imaged after a 2 day growth at 30°C.
(TIFF)

S8 Fig A life-extending dose of TM does not detectably induce microautophagy. Wild type cells harboring a genomically tagged ER membrane protein Sec63 (Sec63-GFP) were treated with the indicated concentrations of TM. Lysates were prepared at the indicated time points and resolved by SDS PAGE. Blots were probed with an anti-GFP antibody.
(TIFF)

S9 Fig Enhanced catabolism of protein aggregates in *rer1Δ*. Reduced level of detergent insoluble protein inclusions in yeast *rer1Δ*. Sarkosyl was added to 2 mg aliquots of total protein lysates from *rer1Δ* and wild type cells before or after a 4hr exposure to TM. Lysates were spun at 1kg to remove cell debris and detergent insoluble inclusions were recovered by centrifuging at 20 kg for 1hr, resolved by SDS-PAGE, and analyzed by coomassie blue staining. 10 μg aliquots of total lysates were analyzed in parallel. A strain harboring a temperature sensitive allele of the molecular chaperone hsp82 (*hsp82* ^*ts*^) served as an experimental control. Inactivating Hsp82 at 37°C leads to a pronounced increase in the level of insoluble protein inclusions.
(TIFF)

S10 Fig Mitotic lifespan in yeast Golgi mannosyltransferase mutant *mnt3Δ*. **A.** High basal expression of *LacZ* from a *KAR2* promoter in *mnt3Δ* mutants. Data represent means of 2 experiments ± s.e.m. **B.** Live cell imaging of GFP-Atg8. Note the increase in the number of autophagosomes in *mnt3Δ*. **C**. Mitotic lifespan of wild type yeast and the isogenic mutants grown on YPD at 30°C. The differences between WT (20.5 days) and the *mnt3∆* (35.5), *rer1∆* (39.5) and *rer1∆ mnt3∆* (34.5) mean mitotic lifespans were significant (p-values<0.001).
(TIFF)

S11 Fig Live cell imaging of GFP-Atg8 in wild type and *ire1Δ* mutants. The average number of GFP foci per cell, scored from Z-stacked images, are plotted (n = 25).
(TIFF)
