# Supplementary figures and images for: A Genome Scale Screen for Mutants with Delayed Exit from Mitosis: Ire1-Independent Induction of Autophagy Integrates ER Homeostasis into Mitotic Lifespan
Source: PLoS Genet. 2015 Aug 6;11(8):e1005429. doi: 10.1371/journal.pgen.1005429 (PMC4527830; doi:10.1371/journal.pgen.1005429)

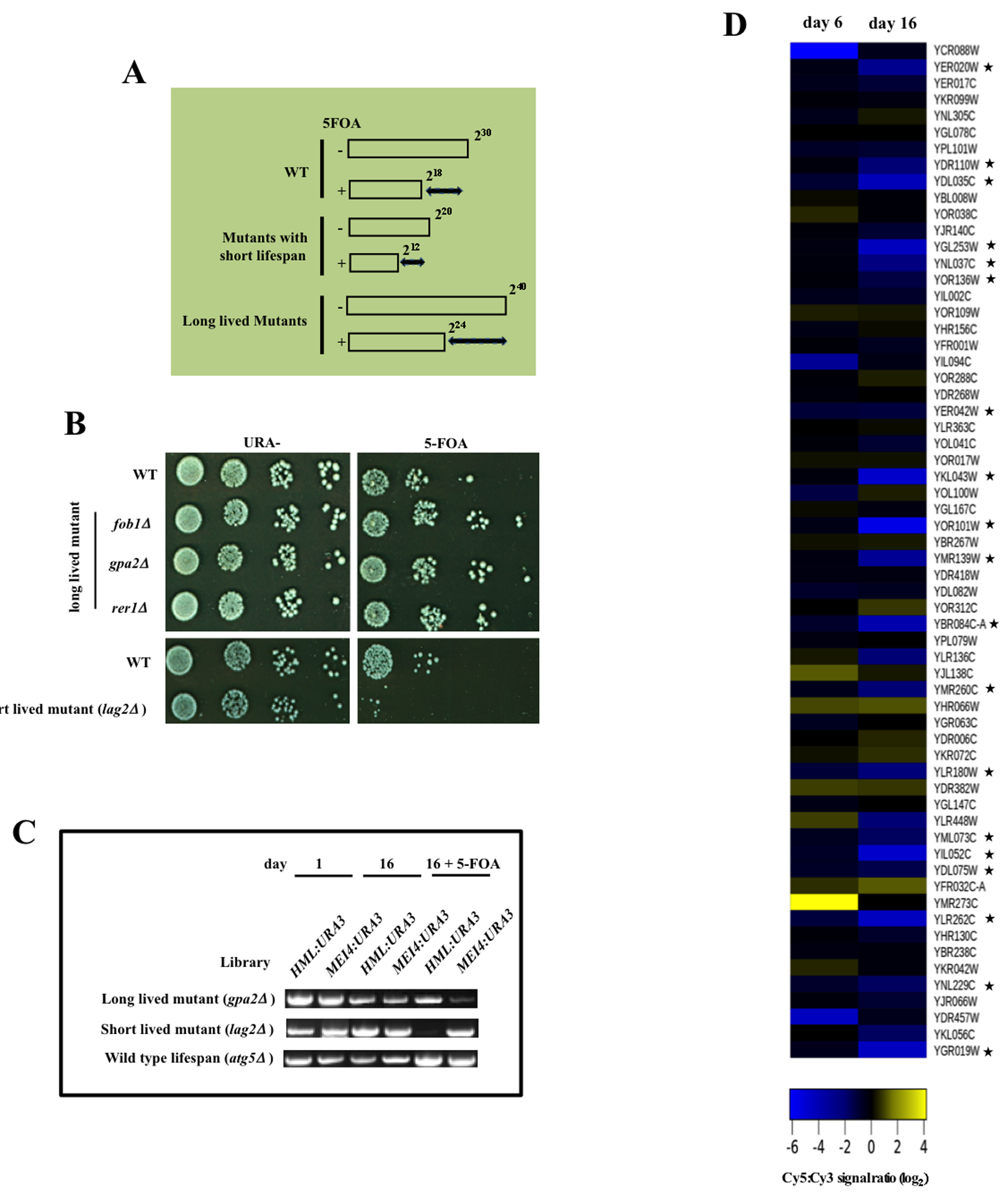

Supplement: S1 Fig — In yeast undergoing early mitotic divisions, the two repositories of the mating type information, HMR and HML, are maintained in a heterochromatic state and are transcriptionally silent [49]. In late mitotic divisions, progressive loss of heterochromtic state leads to loss of transcriptional silencing at the mating loci. We replaced the HML locus in a pool of yeast single deletion mutants with the tractable marker orotidin-5'-phosphate decarboxylase (URA3). Cells that display loss of silencing at the HML locus are selected against using 5-fluoroorotic acid (5-FOA), a cytotoxic uracil analog that inhibits growth of cells expressing URA3. The age-dependent derepression of the mating loci occurs at random during the latter cell divisions (arrows), typically in cells that have completed between 70 and 100 percent of their mitotic divisions [9]. For instance wild type yeast with an average lifespan of 30 generations give rise to a population of 230 daughter cells in the absence of 5-FOA, whereas in the media containing 5-FOA they randomly generate 221−230 daughters. Importantly, the onset of URA3 expression is governed by the mitotic lifespan intrinsic to each mutant; HML::URA3 mutants with short mitotic lifespan are preferentially depleted from the pool of mutants in the presence of 5-FOA due to an earlier expression of URA3. In contrast mutants with prolonged mitotic lifespan are overrepresented due to the delayed expression of URA3. The screen schematic is outlined in Fig 1A. We started with a collection of haploid deletion strains of 4647 nonessential genes [10]. The kanMX4 deletion cassettes are flanked by UPTAG and DOWNTAG sequences unique to each deleted locus. We replaced the HML locus in the pool of mutants with a URA3 reporter via a one-step integration by homologous recombination. In parallel to the query HML::URA3 library, we also constructed a control library by integrating an identical URA3 reporter at the meiotically induced MEI4 locus (MEI4::URA3). Silenc [file pgen.1005429.s001.tiff]

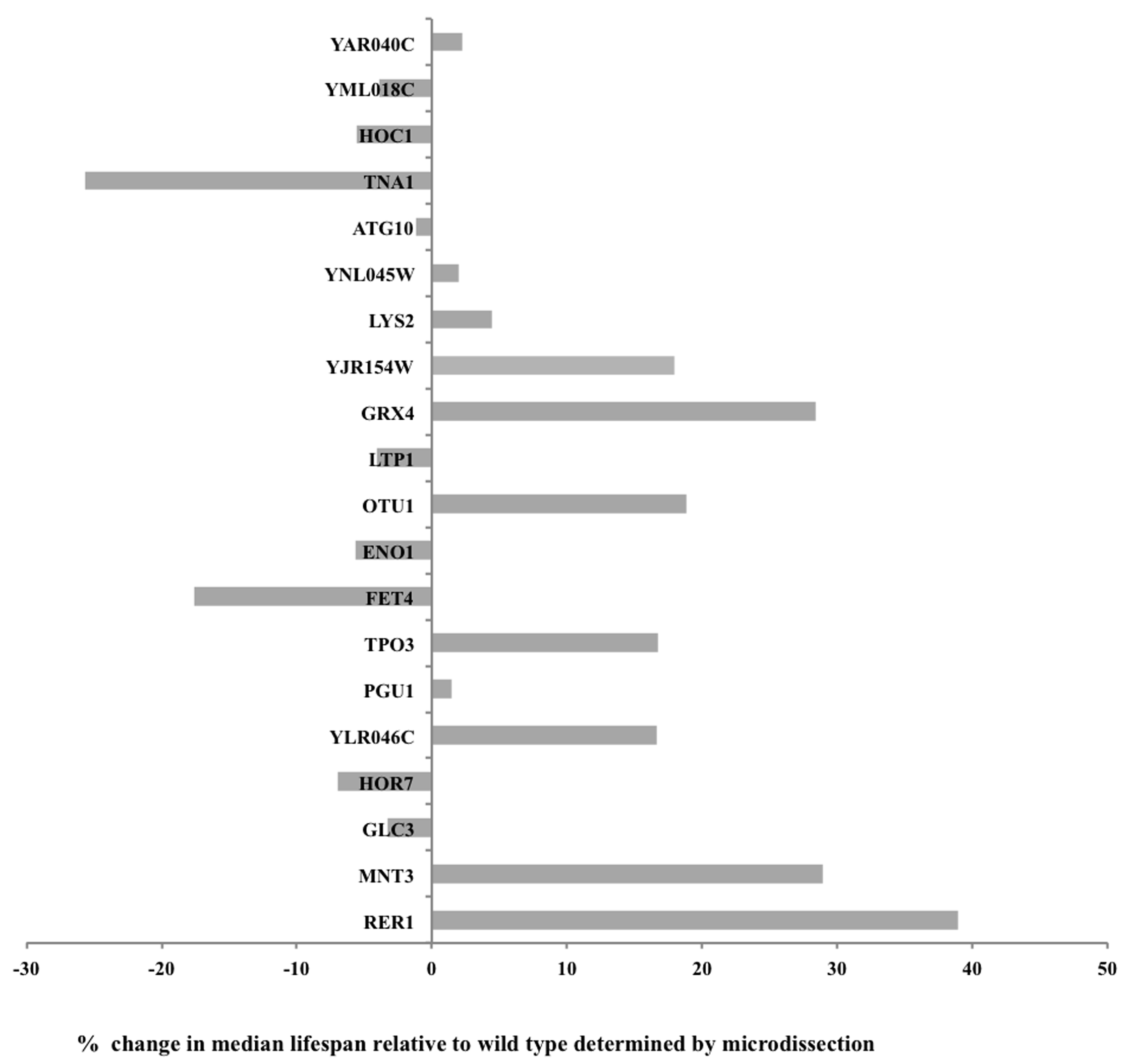

Supplement: S2 Fig — A minimum of 40 mother cells for each strain grown on YPD media at 30°C were assayed by mother-daughter microdissection. Values denote percent change in mean mitotic lifespan relative to the parental wild type assayed in parallel. (TIFF) [file pgen.1005429.s002.tiff]

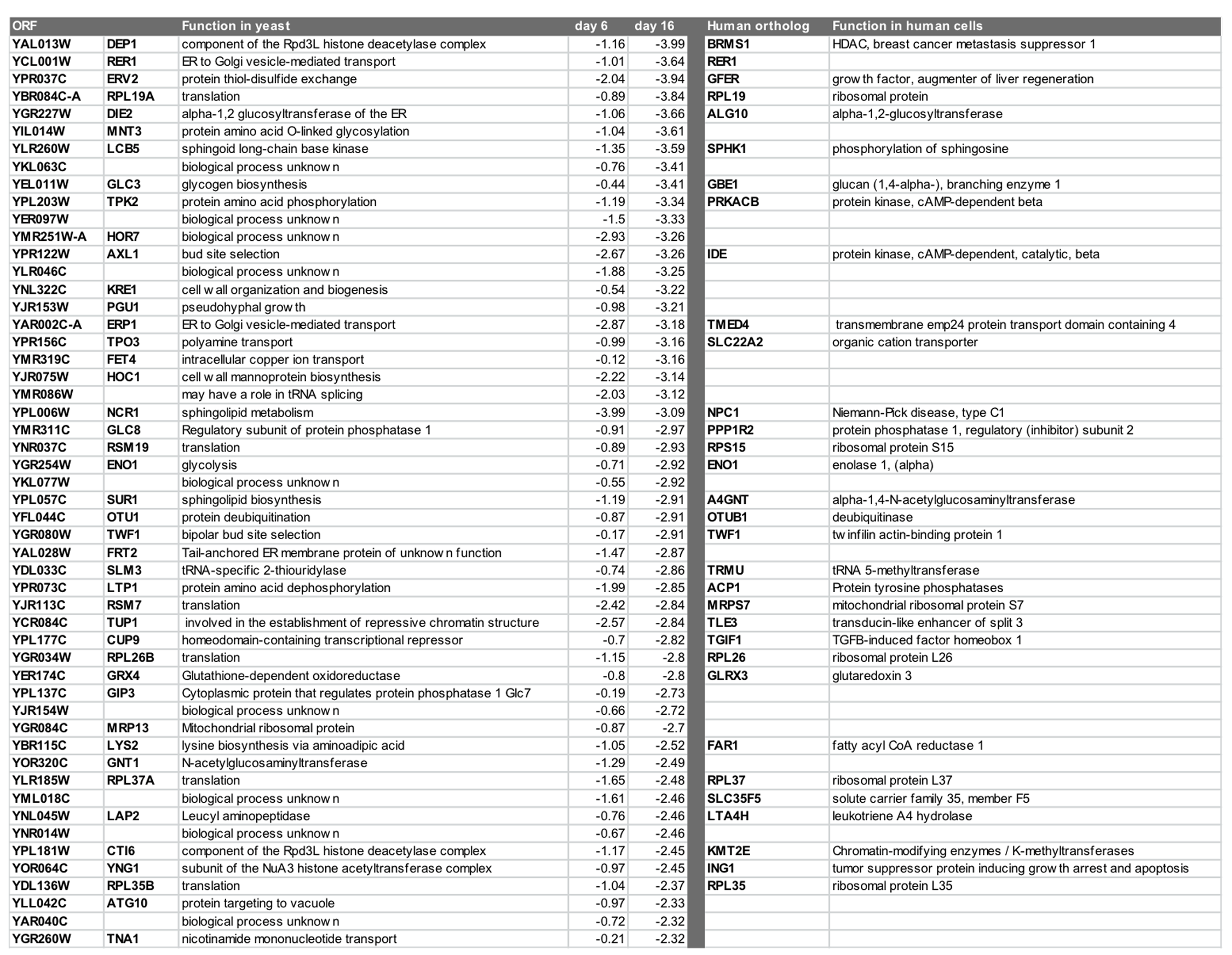

Supplement: S3 Fig — Human functional orthologs are shown. (TIFF) [file pgen.1005429.s003.tiff]

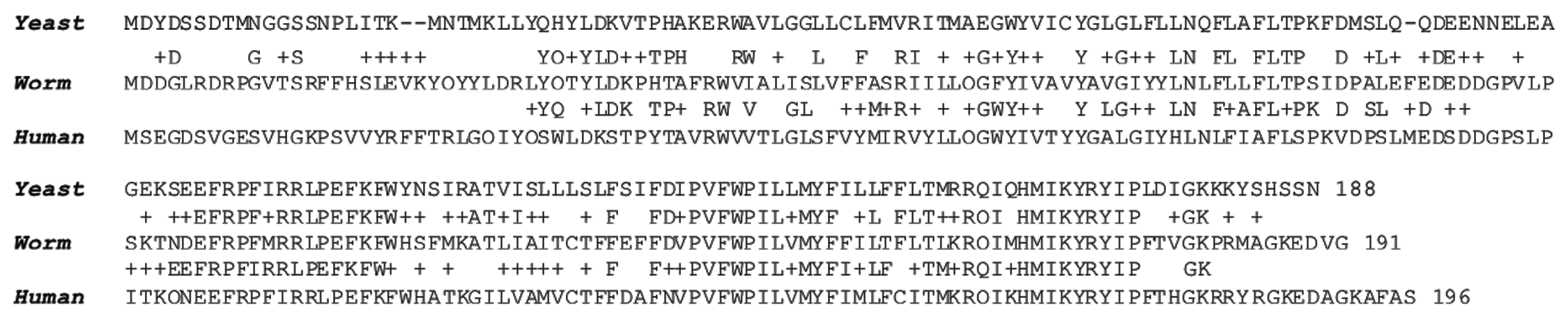

Supplement: S4 Fig — Yeast Rer1 has 51% amino acid identity to human and 48% identity to its C. elegans orthologs. (TIFF) [file pgen.1005429.s004.tiff]

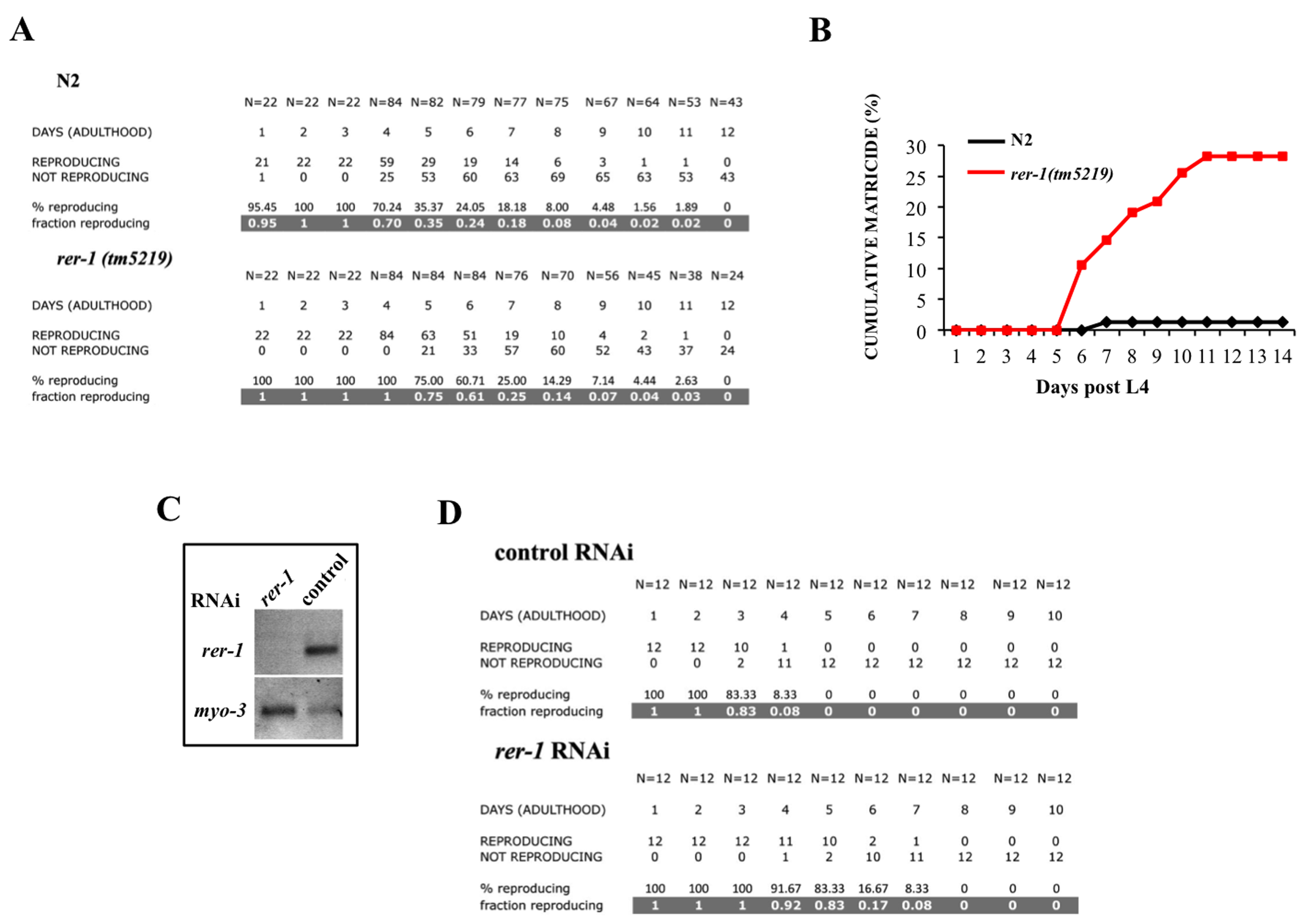

Supplement: S5 Fig — The fraction of reproducing animals was calculated by determining the proportion of adults generating progeny as a function of time. Day 1 corresponds to the first egg-laying day of adulthood (around 9 hours after the L4 lethargus at 20°C). B. Cumulative percentage of matricide in wild type and rer-1(tm5219) mutants. Matricide or “bagging” occurs when fertilized oocytes are retained in the uterus where the embryo and larva ultimately develop. In wild type worms, matricide generally occurs in late stages of reproductive life due to the progressive deterioration of somatic tissues that support egg laying. Unlike wild type worms, matricide levels in rer-1 mutants sharply increased from day 5 onwards, indicating a disconnect between germline and somatic aging. As matricide removes reproductively active animals from the population, it leads to an underestimation of the reproductive lifespan as well as the number of progeny generated in rer-1 mutants. rer-1 expression (C) and reproductive lifespan (D) in wild type hermaphrodites treated with control (empty vector) or rer-1 RNAi. Experiments were performed at 16°C using the rrf-3(pk1426) sensitized background. (TIFF) [file pgen.1005429.s005.tiff]

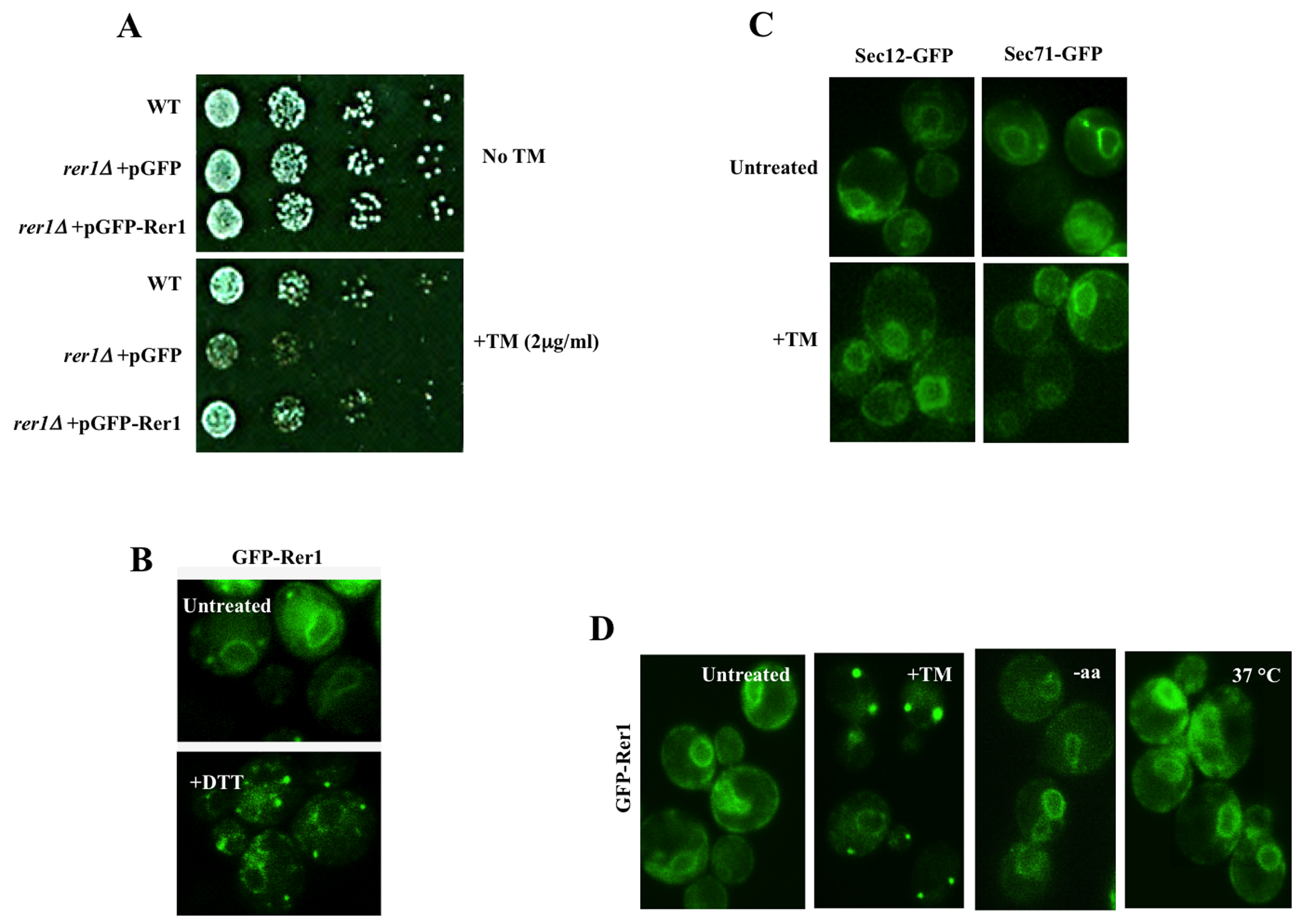

Supplement: S6 Fig — A. Growth of rer1Δ carrying a CEN plasmid expressing an N-terminal GFP fusion of Rer1 (GFP-Rer1) or GFP alone with or without TM. Fivefold serial dilutions of yeast cells were spotted and colonies imaged after a 2 day growth at 30°C. GFP-Rer1, but not GFP alone, restored growth in rer1Δ mutants in the presence of TM. B. GFP-Rer1 relocalization to Golgi after a 2hr treatment with 2mM of the ER stressor DTT. C. Fluorescent images of the GFP fusion of ER proteins Sec71 and Sec12 in normal growth and after a 4 hour exposure to TM. D. Live cell fluorescent images of GFP-Rer1 in cells treated with TM for 2 hours, grown in SC media without amino acids (-aa) or heat shocked at 37°C for 6 hours. GFP-Rer1maintained ER localization in all but TM treated cells. (TIFF) [file pgen.1005429.s006.tiff]

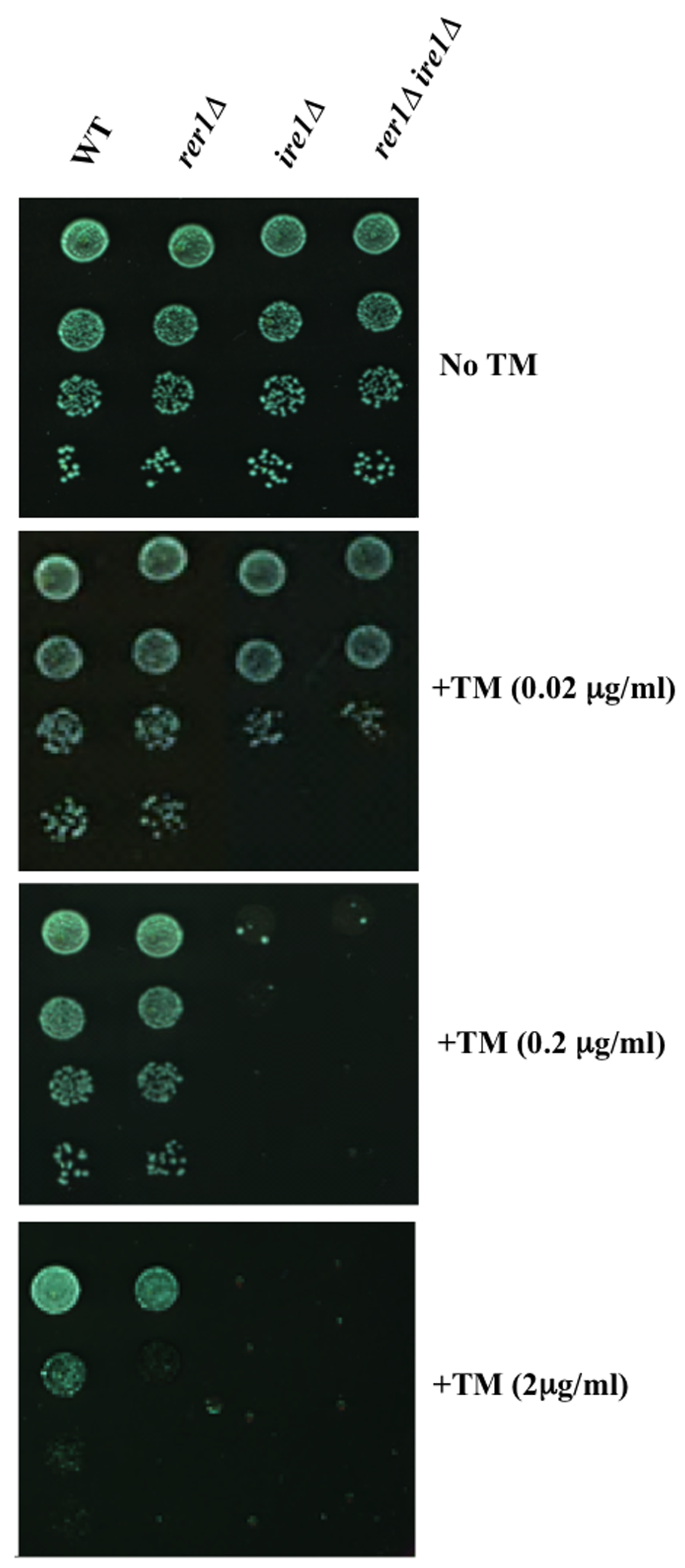

Supplement: S7 Fig — Fivefold serial dilutions of cells were spotted on plates containing the indicated amounts of TM. Colonies were imaged after a 2 day growth at 30°C. (TIFF) [file pgen.1005429.s007.tiff]

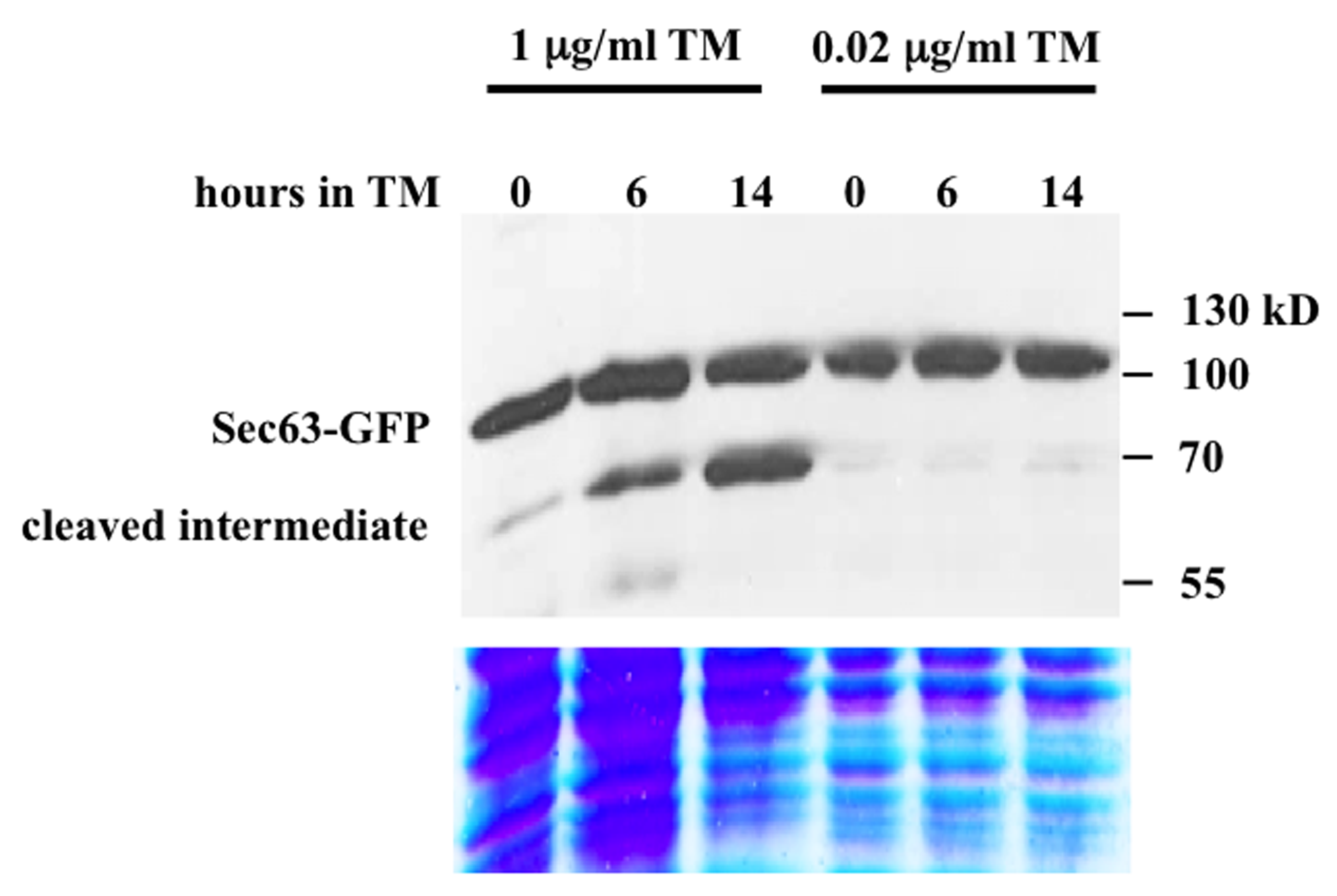

Supplement: S8 Fig — Wild type cells harboring a genomically tagged ER membrane protein Sec63 (Sec63-GFP) were treated with the indicated concentrations of TM. Lysates were prepared at the indicated time points and resolved by SDS PAGE. Blots were probed with an anti-GFP antibody. (TIFF) [file pgen.1005429.s008.tiff]

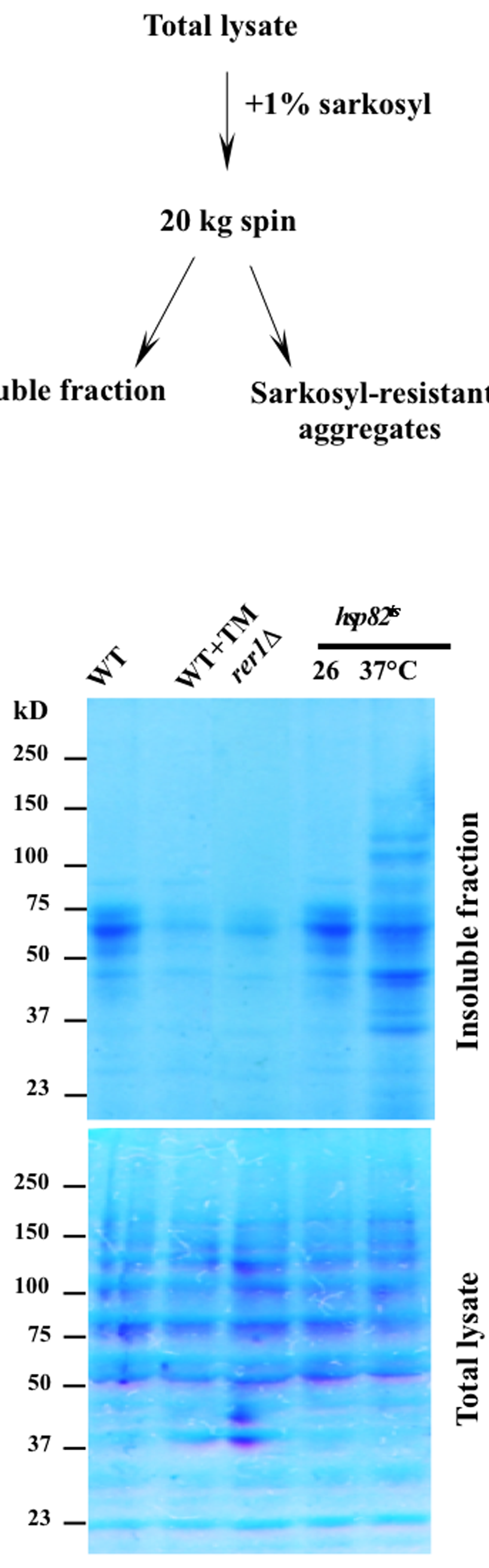

Supplement: S9 Fig — Reduced level of detergent insoluble protein inclusions in yeast rer1Δ. Sarkosyl was added to 2 mg aliquots of total protein lysates from rer1Δ and wild type cells before or after a 4hr exposure to TM. Lysates were spun at 1kg to remove cell debris and detergent insoluble inclusions were recovered by centrifuging at 20 kg for 1hr, resolved by SDS-PAGE, and analyzed by coomassie blue staining. 10 μg aliquots of total lysates were analyzed in parallel. A strain harboring a temperature sensitive allele of the molecular chaperone hsp82 (hsp82 ts) served as an experimental control. Inactivating Hsp82 at 37°C leads to a pronounced increase in the level of insoluble protein inclusions. (TIFF) [file pgen.1005429.s009.tiff]

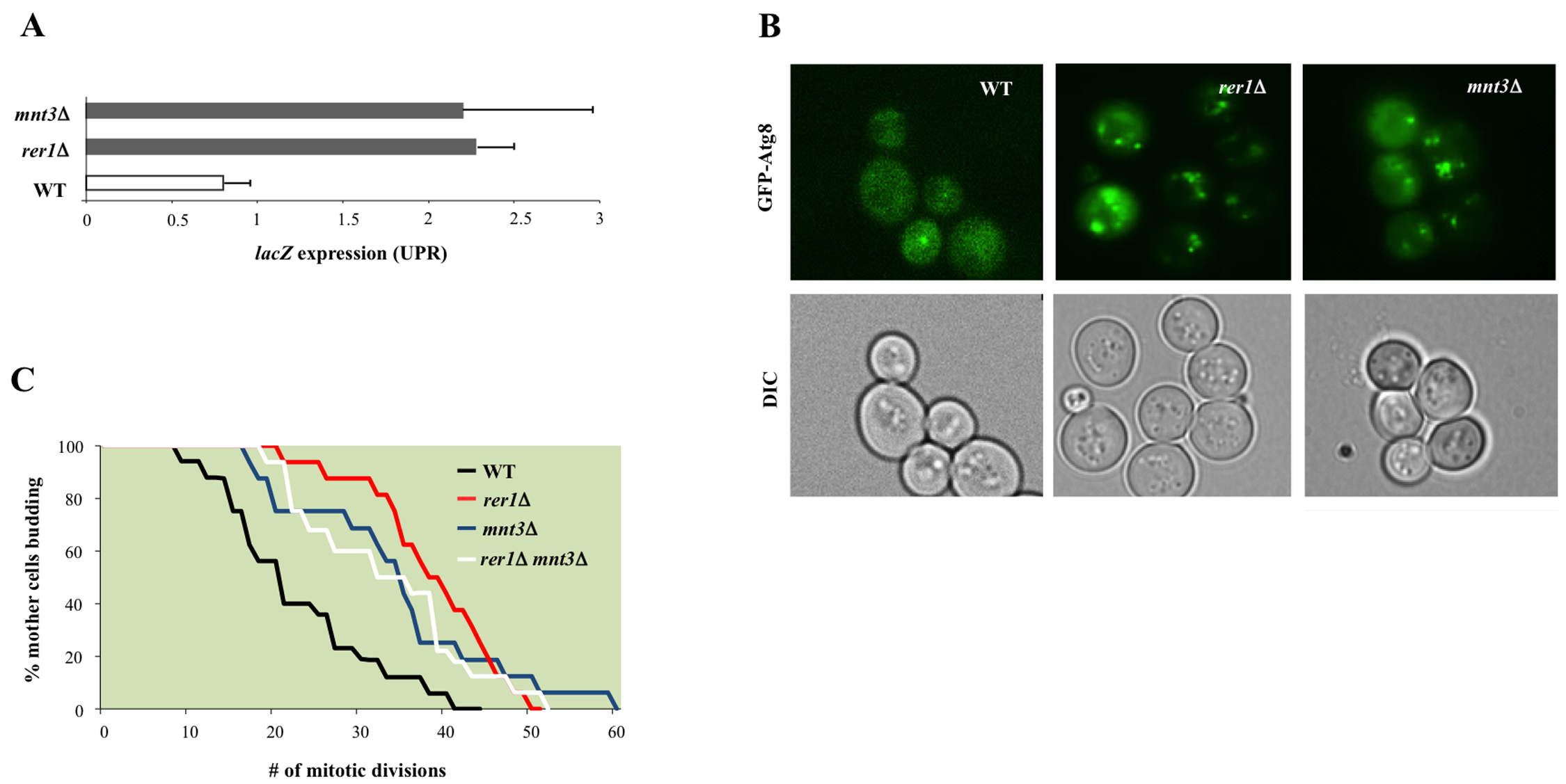

Supplement: S10 Fig — A. High basal expression of LacZ from a KAR2 promoter in mnt3Δ mutants. Data represent means of 2 experiments ± s.e.m. B. Live cell imaging of GFP-Atg8. Note the increase in the number of autophagosomes in mnt3Δ. C. Mitotic lifespan of wild type yeast and the isogenic mutants grown on YPD at 30°C. The differences between WT (20.5 days) and the mnt3∆ (35.5), rer1∆ (39.5) and rer1∆ mnt3∆ (34.5) mean mitotic lifespans were significant (p-values<0.001). (TIFF) [file pgen.1005429.s010.tiff]

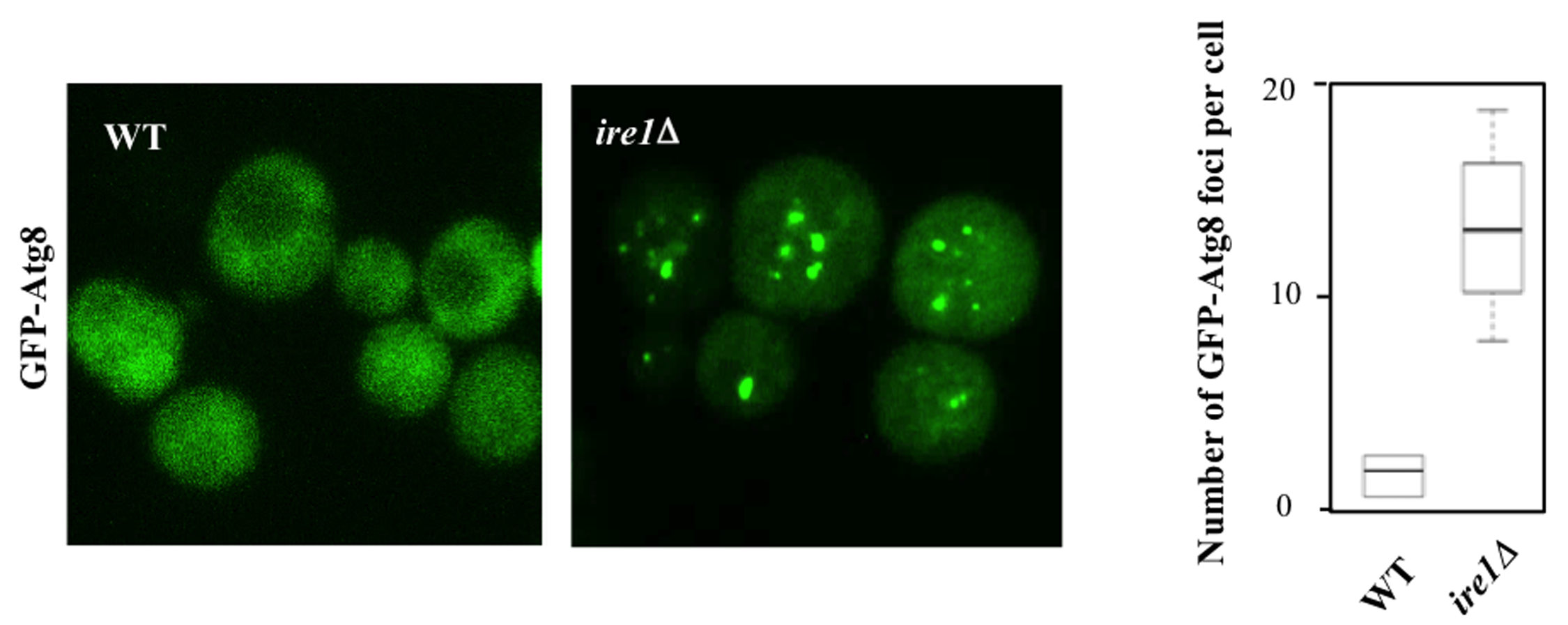

Supplement: S11 Fig — The average number of GFP foci per cell, scored from Z-stacked images, are plotted (n = 25). (TIFF) [file pgen.1005429.s011.tiff]
